# The differential measure for Pythagorean fuzzy multiple criteria group decision-making

**DOI:** 10.1007/s40747-022-00913-4

**Published:** 2022-12-07

**Authors:** Iman Mohamad Sharaf

**Affiliations:** Department of Basic Sciences, Higher Technological Institute, Tenth of Ramadan City, Egypt

**Keywords:** Multi-criteria group decision-making, Pythagorean fuzzy sets, Information measures, Similarity measures, Distance measures, Photovoltaic cells

## Abstract

Pythagorean fuzzy sets (PFSs) proved to be powerful for handling uncertainty and vagueness in multi-criteria group decision-making (MCGDM). To make a compromise decision, comparing PFSs is essential. Several approaches were introduced for comparison, e.g., distance measures and similarity measures. Nevertheless, extant measures have several defects that can produce counter-intuitive results, since they treat any increase or decrease in the membership degree the same as the non-membership degree; although each parameter has a different implication. This study introduces the differential measure (DFM) as a new approach for comparing PFSs. The main purpose of the DFM is to eliminate the unfair arguments resulting from the equal treatment of the contradicting parameters of a PFS. It is a preference relation between two PFSs by virtue of position in the attribute space and according to the closeness of their membership and non-membership degrees. Two PFSs are classified as identical, equivalent, superior, or inferior to one another giving the degree of superiority or inferiority. The basic properties of the proposed DFM are given. A novel method for multiple criteria group decision-making is proposed based on the introduced DFM. A new technique for computing the weights of the experts is developed. The proposed method is applied to solve two applications, the evaluation of solid-state drives and the selection of the best photovoltaic cell. The results are compared with the results of some extant methods to illustrate the applicability and validity of the method. A sensitivity analysis is conducted to examine its stability and practicality.

## Introduction

Globalization, internationalization, and cross-border activities can have a great impact on the decision-making processes of managers responsible for making these complex interrelated decisions [1]. Managers are under continuous pressure to account for the challenges and opportunities that exist in such a competitive environment [2]. They need to address all the issues associated with their decisions. This task can have a direct impact on the effectiveness as well as the efficiency of the decision-making processes [1]. Moreover, in the analysis of practical decision problems, it is difficult to meet the requirements in dealing with complex decision-making problems by expressing the preferences of experts through accurate values [3].

Multiple criteria decision‐making (MCDM) is a branch of operations research and an advanced mathematical tool that explicitly evaluates the feasible alternatives over conflicting multiple criteria in decision‐making to identify the optimal solution [4]. As it is usually challenging for a single individual to take all the sides of a problem into full consideration due to the increasing complexity of decision contexts, group decision-making (GDM) becomes necessary [5]. Group decision‐making (GDM) is a process in which multiple individuals interact simultaneously to analyze problems, evaluate the possible available alternatives characterized by multiple conflicting criteria, and choose a suitable alternative solution to the problem [4]. In consequence, multiple criteria group decision-making techniques (MCGDM) have been proposed to consider the opinion of different decision-makers/managers with distinct skills and varied knowledge [6].

The fuzzy theory was first introduced by Zadeh [7] to imitate human reasoning and cognition. It has been widely investigated due to its strong ability to deal with complex nonlinearities and to model systems with uncertain parameters in practical applications, e.g., fault detection for security-critical engineering, for instance, wind turbine, power network, and nuclear industry [8, 9]. It also proved to be successful in handling some of the aforementioned difficulties in the decision processes and became a cornerstone in the development of innovative, robust, and flexible MCDM methods for making better business decisions. It also encouraged researchers to propose several extensions and generalizations of fuzzy sets to cope with the increasing complexity of imprecision and vagueness associated with various practical applications. For example, neutrosophic fuzzy sets [10], hesitant fuzzy sets [11, 12], picture fuzzy sets [13], q-rung orthopair fuzzy sets [14], and spherical fuzzy sets [15–17]. Moreover, several variants of each type have been proposed, e.g., the bipolar neutrosophic sets [18], complex single-valued neutrosophic sets [19], the dual hesitant fuzzy sets [20], and the complex spherical fuzzy sets [21]. Each of these sets has its characteristics, and the employment of these sets in diverse applications depends mainly on the application and the uncertainty and ambiguity of the information. Various MCDM and MCGDM methods that utilize diverse fuzzy sets have been utilized in solving various applications, e.g., economy [22, 23], risk assessment [23, 24], renewable energy [25, 26], green supplier selection [27–29], and health care [30–32]. Even during the COVID-19 pandemic, MCDM methods played a role [33–37].

Intuitionistic fuzzy sets (IFSs) are one of the most prominent extensions. IFSs were initially introduced by Atanassov [38] to generalize the concept of fuzzy sets. Instead of giving the degree of membership only to an element in a given set, an IFS gives a degree of membership and a degree of non-membership. The sum of these two parameters is less than or equal to one. Yager and Abbasov [39] and Yager [40] proposed Pythagorean fuzzy sets (PFSs) as an advanced development of IFSs. The main purpose is to enlarge the value range of the preference information. Similar to IFSs, PFSs are characterized by the same two parameters expressing the degrees of membership and non-membership. However, the sum of the squares of these two parameters is equal to or less than one. Therefore, PFSs provide a wider domain and are more capable of modeling vagueness and uncertainty in various practical applications. PFSs can also interpret human preference and non-preference information in a broader sense.

The suitability of PFSs for expressing ambiguous information has attained many researchers’ attention. Remarkable contributions in the family of multi-criteria decision-making (MCDM) and multi-criteria group decision-making (MCGDM) methods have been made utilizing PF information [41]. Zhang and Xu [42] presented an extension of TOPSIS method in the PF context with applications. Zhang [43] developed a PF hierarchical QUALIFLEX approach with application in risk evaluation of strategic emerging industries. Ren et al. [44] made a case study on selecting the governor of the Asian Infrastructure Investment Bank utilizing a TODIM approach that handles PF-MCGDM problems. Wan et al. [45] proposed a three-phase group decision-making method using PF information with a haze management example. Akram et al. [46] extended the ELECTRE-I method using PFSs in a group decision-making environment with applications in health safety and environment management. Akram et al. [47] developed a two-phase Pythagorean fuzzy version of ELECTRE III method to take full advantage of the capabilities of PFSs.

In the Pythagorean fuzzy environment, measuring the difference between PFSs plays an important role in inference problems [48]. Therefore, several approaches were proposed for estimating the difference. The most salient methods are distance measures and similarity measures.

Similarity measures were introduced as an important and useful tool to find the degree of similarity between two objects. Several functions were proposed to express the degree of similarity between sets and were applied in different applications, e.g., physical anthropology, automatic classification, ecology, psychology, information retrieval, medical diagnosis, and pattern recognition [49]. The similarity measure is one of the research hotspots in fuzzy set theory. It is also known to be an important tool in MCDM. The most commonly applied similarity measures are Jaccard, Dice, and the cosine similarity measures. Up till now, similarity measures determine how much two PFSs are similar or dissimilar, but they do not differentiate the PFSs based on the dissimilarity, i.e., they measure how much they are similar but do not reveal which one is better and to what extent.

Similarity and distance measures are two faces of a coin. Most similarity measures are based on distance measures. Comparing two PFSs depends to a large extent on both measures. Although many studies proposed several distance measures, still extant distance measures have some flaws [50]. First, they can produce counter-intuitive results [48]. Second, they might fail to derive the maximum distance measure value [50]. This will influence the ranking values of the alternatives and produce false results. Therefore, one of the open issues in the Pythagorean fuzzy environment is how to measure the distance between two PFSs [48].

Up to now, the extant distance approaches that have been proposed in the literature compute precise distance measures for PFSs. According to Habib et al. [51], how can we be sure about the distances between PFSs, while we cannot be sure about the sets themselves? Then, it is not acceptable to specify exact distances between PFSs.

The main defect in the extant measures can be attributed to the treatment of the parameters of a PFS equally, although each parameter has a different influence, i.e., an increase in the membership degree has the same influence as an increase in the non-membership degree, although the former is an advantage and the latter is a disadvantage. Hence, it is important to find a discrimination method that preserves the identity of each parameter of a PFS to avoid the deficiencies of both distance and similarity measures that result in counter-intuitive results.

Since the two parameters of a PFS, the membership $\left(\mu \right)$ and the non-membership $\left(\upsilon \right)$ degrees, describe the cognitive uncertainty of the decision-maker with fine granularity, these two elements can be treated as two typical attributes representing $\left(\mu \right)$ on the horizontal axis and $\left(\upsilon \right)$ on the vertical axis on Cartesian coordinates [52]. In this attribute space, a new concept is proposed to differentiate PFSs, the differential measure (DFM).

In this article, the concept of differential measure (DFM) is introduced as a new approach for comparing PFSs. It is a preference relation between two PFSs by virtue of position in the attribute space and according to the closeness of their membership and non-membership degrees. The extant distance measures and similarity measures have several drawbacks due to the equal treatment of the membership (support) and non-membership degree (opposition), although each direction has a different implication. The DFM utilizes signed distance to account for the impact of each component of a Pythagorean fuzzy assessment. An increment in the support direction is considered a positive step, while an increment in the opposition direction is considered a negative step. Since similarity measures indicate how much two PFSs are similar and do not reveal which one is better and, to what extent, the DFM can better indicate the discrimination degree of PFSs when making a decision. Two Pythagorean fuzzy sets (PFSs) are classified as identical, equivalent, superior, or inferior to one another, giving the degree of superiority or inferiority. A new method for MCGDM is proposed based on the introduced DFM. Furthermore, a new technique for computing the weights of the experts is developed. Two practical problems, namely, the evaluation of solid-state drives and the selection of the best photovoltaic cell, are solved in the Pythagorean fuzzy environment using the proposed method. Then, the applicability and feasibility of the model developed in this article are demonstrated by comparison with some existing techniques. Additionally, a sensitivity analysis is conducted to illustrate its stability and practicality. Consequently, the contribution of this study is triple manifold.

Since PFSs are more flexible than IFSs to deal with absurdness and uncertainty and to cope with human evaluation information, it is necessary to pay more attention to group decision‐making in this context. Therefore

- The concept of DFM is introduced as a new approach for comparing PFSs. It is a preference relation between two PFSs according to their position in the attribute space. A DFM preserves the identity of the parameters of PFSs to avoid the drawbacks of the extant distance and similarity measures in discrimination.
- A new MCGDM method is introduced based on the proposed DFM.
- A new technique for computing the experts’ weights in MCGDM problems is proposed. This technique can also yield a solution to the MCGDM problem.

The article is organized as follows. In the section “A literature review”, the recently proposed information measures and comparison methods of PFSs are reviewed. The basic concepts of PFSs are given in the section “Preliminaries”. The new concept of DFM is introduced in the section “The differential measure” with a new MCGDM method based on the concept. A novel technique for computing the experts’ weights is introduced in the section “Determining the experts’ weights”. Two decision-making problems are solved with the proposed MCGDM method in the section “Practical examples”. A sensitivity analysis is carried out in the section “The sensitivity analysis”. In the section “Conclusion and discussion”, the conclusion is given with the direction of future research.

## A literature review

Researchers classified the methods of comparing PFSs to information measures and comparison methods. Information measures encompass several approaches, such as distance measures, similarity measures, entropy, inclusion measures, divergence measure, and correlation coefficients [53, 54]. Comparison methods include score functions, accuracy functions, closeness index, and comparison value [55]. While information measures are exploited in evaluation and assessment, comparison measures are mainly utilized in ranking.

### The information measures

The information measures have played a crucial role in the development of PFSs theory and its applications [53]. Peng et al. [56] constructed the axiomatic definitions of the Pythagorean fuzzy information measures and presented the corresponding formulas, and discussed their transformation relationships.

The similarity measures between fuzzy sets attracted researchers’ attention due to their wide applications in various fields, such as machine learning, decision-making, image processing, and pattern recognition [57]. The similarity measures are used mainly to differentiate between different fuzzy sets. Most of the proposed similarity measures are derived from distance measures.

Zhang [58] developed a novel decision method based on a new similarity measure to address MCDM problems in the Pythagorean fuzzy environment. Peng et al. [56] investigated the relationship between the distance measures, the similarity measures, the entropy, and the inclusion measures for PFSs. They proposed some information measures for PFSs with their applications. Qin et al. [53] introduced new distance measures for PFSs and employed them in a MCDM application. Peng and Dai [59] initiated a new axiomatic definition of the Pythagorean fuzzy distance measure which is expressed by a PFS reducing the information loss and retaining more original information.

Wei and Wei [57] proposed ten similarity measures between two PFSs using the cosine function and applied these similarity measures to pattern recognition and medical diagnosis. Biswas and Sarkar [60] proposed a series of similarity measures based on point operators for Pythagorean fuzzy sets. Chen [61] presented a flexible and multipurpose definition of a distance measure for PFSs based on the Minkowski distance model. Li and Zeng [62] proposed several distance measures for PFSs which takes into account the four parameters that can be used to define a PFS, namely, the degree of membership, the degree of non-membership, the strength of commitment, and the direction of commitment. Zeng et al. [55] also proposed a variety of distance measures incorporating five parameters this time, the previous four parameters together with the indeterminacy degree. They also presented some similarity measures based on these distance measures.

Wang et al. [49] presented some novel Dice similarity measures for PFSs to handle pattern recognition, citation analysis, information retrieval, and multiple attribute decision-making. Hussian and Yang [63] proposed some distance measures based on the Hausdorff metric and developed some similarity measures using these distance measures. Xiao and Ding [48] proposed a novel distance measure between PFSs that exploits the Jensen–Shannon divergence. Ejegwa [64] proposed some distance and similarity measures that satisfy metric conditions in the Pythagorean fuzzy environment. Firozja et al. [65] proposed a similarity measure using triangular conorms. Huang et al. [50] proposed two novel distance measures for computing the deviation degree between two PFSs. Türkarslan et al. [66] proposed similarity measures based on the Choquet integral using the trigonometric functions cosine and cotangent. Habib et al. [51] examined the use of Pythagorean fuzzy distances and similarity measures in a minimum spanning tree agglomerative hierarchical clustering method.

The Pythagorean fuzzy entropy (PFE) is used to measure fuzziness and uncertainty occurring in a PFS. High entropy indicates more information in the process; a high entropy value represents low uncertainty. The PFE is mainly used in Pythagorean fuzzy MCDM to determine the objective weights of the criteria which are essential in the evaluation of alternatives. Peng et al. [56] proposed a PFE relying on the axiomatic definition of Burillo and Bustince [67] of entropy for IFSs. Wan et al. [45] defined a PFE as inspired by the entropy definition of IFSs introduced by Szmidt and Kacprzyk [68]. Xue et al. [69] proposed a PFE grounded on the similarity and hesitancy parts where the similarity part and the hesitancy part reflect the fuzziness and uncertainty of PFSs, respectively. Yang and Hussain [70] proposed new PFEs based on probability type, distance, Pythagorean index, and min–max operation. Gandotra et al. [71] proposed a new entropy measure for PFSs determined by the notion of Wang et al. [72] in the context of IFSs.

Garg [73] introduced the correlation measures for PFSs with their applications in pattern recognition and medical diagnosis. Thao [74] developed a formula for calculating the correlation coefficient based on the variance and covariance of PFSs. Due to some flaws in these correlation coefficients, Singh and Ganie [54] proposed novel correlation measures that avoid these flaws and applied these measures to pattern recognition, medical diagnosis, MCDM, and clustering analysis. Ejegwa [75] proposed a tri-parametric correlation coefficient, a generalization of Garg’s correlation coefficient, for a better output in resolving MCDM problems in the Pythagorean fuzzy environment. Ejegwa and Jana [76] improved Garg’s methods of computing weighted correlation coefficients for PFSs to be more reliable with better performance indices than the existing ones.

The Pythagorean fuzzy inclusion measure also called the Pythagorean fuzzy subset hood measure indicates the degree to which one PFS is contained in another PFS. Peng et al. [56] constructed the axiomatic definitions of the Pythagorean fuzzy inclusion measure and presented the corresponding formulas. Mandal and Ranadive [77] provided an inclusion measure for PFSs based on the theorems presented by Peng et al. [56].

Xiao and Ding [48] considered the discrepancy of data from the perspective of relative entropy. They proposed a novel divergence measure between PFSs taking advantage of the Jensen–Shannon entropy measure. Agarwal [78] proposed a flexible and generalized parametric divergence measure of order $\alpha$ and degree $\beta$ denoted as a class of $\left(\alpha ,\beta \right)$.

### Comparison methods

Four comparison methods have been presented to compare PFSs [55]: score functions, score and accuracy functions, closeness index, and comparison value.

Zhang and Xu [42] defined a score function for PFSs and provided a comparison law based on this score function. Ma and Xu [79] defined another score function utilizing the square root. Peng and Dai [59] proposed a new score function employing Euler’s constant. Peng [80] proposed a score function using Euler’s constant. Yet, all the proposed score functions produce equal score function values for different PFSs. Therefore, the accuracy function of Zhang [58] is always employed in situations where score functions are invalid. To overcome the defects of the existing score functions, Huang et al. [50] developed a novel score function utilizing the logarithmic function based on both determinacy and indeterminacy degrees.

Zhang [43] proposed the concept of closeness index for PFSs based on distance measures of PFSs and presented a new ranking method utilizing this new closeness index. Akram et al. [4] developed a revised closeness index to obtain the ranking of alternatives and to identify the optimal alternative within a step-wise PF‐TOPSIS method for MCGDM.

Yager [40] introduced a scalar formula for comparing PFSs and proposed comparison laws based on this formula.

It has been observed from the above studies that almost all the proposed methods for comparing PFSs produce crisp values, regardless of the information structure of a PFS that contains two contradicting pairs. As a result, several drawbacks might arise in some cases which will influence the ranking values of the alternatives whenever PFSs are used to express the values of the criteria. The main aim of the study is to propose a new measure that can better discriminate PFSs and avoid generating counter-intuitive results while preserving their information structure to reduce information loss and retain more original information. This, in turn, will increase the validity and reliability of the obtained results.

## Preliminaries

### Definition 1

[40]. A PFS $\widetilde{A}$ in a finite universe of discourse $X$ is represented by

1 $$\widetilde{A}=\left\{x,{\mu }_{\widetilde{A}}\left(x\right),{\upsilon }_{\widetilde{A}}\left(x\right):x\in X\right\},$$

where ${\mu }_{\widetilde{A}}\left(x\right):X\to [\mathrm{0,1}]$ denotes the membership degree, ${\upsilon }_{\widetilde{A}}\left(x\right):X\to [\mathrm{0,1}]$ denotes the non-membership degree, satisfying the condition

2 $${0\le \mu }_{\widetilde{A}}^{2}\left(x\right)+{\upsilon }_{\widetilde{A}}^{2}\left(x\right)\le 1.$$

The degree of hesitation, i.e., the degree of indeterminacy, is represented by

3 $${\pi }_{\widetilde{A}}\left(x\right)=\sqrt{1-\left({\mu }_{\widetilde{A}}^{2}\left(x\right)+{\upsilon }_{\widetilde{A}}^{2}\left(x\right)\right)}.$$

### Definition 2

[42]. For any two PFSs $\widetilde{A}=\left({\mu }_{\widetilde{A}},{\upsilon }_{\widetilde{A}}\right)$ and $\widetilde{B}=\left({\mu }_{\widetilde{B}},{\upsilon }_{\widetilde{B}}\right)$, the operational laws are as follows:

(i) 4 $$\widetilde{A}\oplus \widetilde{B}=\left(\sqrt{{\mu }_{\widetilde{A}}^{2}+{\mu }_{\widetilde{B}}^{2}-{\mu }_{\widetilde{A}}^{2}{\mu }_{\widetilde{B}}^{2}},{\upsilon }_{\widetilde{A}}{\upsilon }_{\widetilde{B}}\right),$$
(ii) 5 $$\widetilde{A}\otimes \widetilde{B}=\left({\mu }_{\widetilde{A}}{\mu }_{\widetilde{B}},\sqrt{{\upsilon }_{\widetilde{A}}^{2}+{\upsilon }_{\widetilde{B}}^{2}-{\upsilon }_{\widetilde{A}}^{2}{\upsilon }_{\widetilde{B}}^{2}}\right),$$
(iii) 6 $$\lambda \odot \widetilde{A}=\left(\sqrt{1-{\left(1-{\mu }_{\widetilde{A}}^{2}\right)}^{\lambda }},{\upsilon }_{\widetilde{A}}^{\lambda }\right),$$
(iv) 7 $${\widetilde{A}}^{\lambda }=\left({\mu }_{\widetilde{A}}^{\lambda },\sqrt{1-{\left(1-{\upsilon }_{\widetilde{A}}^{2}\right)}^{\lambda }}\right),\mathrm{where } \; \lambda >0 \; \mathrm{ is \;  a \; scalar}.$$

### Definition 3

[40]. For a set of PFSs $\widetilde{\mathbf{A}}=\left\{{\widetilde{A}}_{1},{\widetilde{A}}_{2},\dots ,{\widetilde{A}}_{n}\right\}$ with weights $\left({w}_{1},{w}_{2}, \dots ,{w}_{n}\right)$, where ${w}_{i}\in \left[\mathrm{0,1}\right]$ and $\sum_{i=1}^{n}{w}_{i}=1$, the Pythagorean fuzzy weighted averaging operator (PFWA_*Y*_) and the Pythagorean fuzzy weighted geometric operator (PFWG_*Y*_) are defined by

(i) 8 $${\mathrm{PFWA}}_{Y}\left\{{\widetilde{A}}_{1},{\widetilde{A}}_{2},\dots ,{\widetilde{A}}_{n}\right\} ={w}_{1}{\widetilde{A}}_{1}+{w}_{2}{\widetilde{A}}_{2}+\dots +{w}_{n}{\widetilde{A}}_{n}=\left(\sum_{i=1}^{n}{w}_{i}{\mu }_{{\widetilde{A}}_{i}},\sum_{i=1}^{n}{w}_{i}{\upsilon }_{{\widetilde{A}}_{i}} \right),$$
(ii) 9 $${\mathrm{PFWG}}_{Y}\left\{{\widetilde{A}}_{1},{\widetilde{A}}_{2},\dots ,{\widetilde{A}}_{n}\right\} ={\widetilde{A}}_{1}^{{w}_{1}}+{\widetilde{A}}_{2}^{{w}_{2}}+\dots +{\widetilde{A}}_{n}^{{w}_{n}}=\left(\prod_{i=1}^{n}{\mu }_{{\widetilde{A}}_{i}}^{{w}_{i}},\prod_{i=1}^{n}{\upsilon }_{{\widetilde{A}}_{i}}^{{w}_{i}} \right).$$

### Definition 4

[42]. The score function of a PFS $\widetilde{A}$ is given by

10 $${{\mathrm{Sc}}_{ZX}\left(\widetilde{A}\right)=\mu }_{\widetilde{A}}^{2}\left(x\right)-{\upsilon }_{\widetilde{A}}^{2}\left(x\right),$$

where. $${\mathrm{Sc}}_{ZX}\left(\widetilde{A}\right)\in \left[-\mathrm{1,1}\right]$$

### Definition 5

[81]. The accuracy function of a PFS $\widetilde{A}$ is given by

11 $${{\mathrm{Ac}}_{\mathrm{PY}}\left(\widetilde{A}\right)=\mu }_{\widetilde{A}}^{2}\left(x\right)+{\upsilon }_{\widetilde{A}}^{2}\left(x\right),$$

### Definition 6

[73]. The complement of a PFS $\widetilde{A}=\left({\mu }_{\widetilde{A}},{\upsilon }_{\widetilde{A}}\right)$ is given by

12 $${\widetilde{A}}^{c}=\left({\upsilon }_{\widetilde{A}},{\mu }_{\widetilde{A}}\right).$$

Hussian and Yang [63] and Xiao and Ding [48] concurrently extended the normalized Hamming distance and the normalized Euclidean distance in the Pythagorean fuzzy environment as follows

### Definition 7

[48, 63]. The normalized Hamming distance between two PFSs $\widetilde{\mathbf{A}}$ and $\widetilde{\mathbf{B}}$ is given by

13 $${d}_{\mathrm{NH} }\left(\widetilde{\mathbf{A}},\widetilde{\mathbf{B}}\right)=\frac{1}{2n}\sum_{i=1}^{n}\left\{\left|{\mu }_{\widetilde{A}}^{2}\left({x}_{i}\right)-{\mu }_{\widetilde{B}}^{2}\left({x}_{i}\right)\right|+\left|{\upsilon }_{\widetilde{A}}^{2}\left({x}_{i}\right)-{\upsilon }_{\widetilde{B}}^{2}\left({x}_{i}\right)\right|+\left|{\pi }_{\widetilde{A}}^{2}\left({x}_{i}\right)-{\pi }_{\widetilde{B}}^{2}\left({x}_{i}\right)\right|\right\}.$$

### Definition 8

[48, 63] The normalized Euclidean distance between two PFSs is given by

14 $${d}_{\mathrm{NE}}\left(\widetilde{\mathbf{A}},\widetilde{\mathbf{B}}\right)=\sqrt{\frac{1}{2n} {\sum\nolimits_{{i = 1}}^{n} }{\left({\mu }_{\widetilde{A}}^{2}\left({x}_{i}\right){-\mu }_{\widetilde{B}}^{2}\left({x}_{i}\right)\right)}^{2}+{\left({\upsilon }_{\widetilde{A}}^{2}\left({x}_{i}\right)-{\upsilon }_{\widetilde{B}}^{2}\left({x}_{i}\right)\right)}^{2}+{\left({\pi }_{\widetilde{A}}^{2}\left({x}_{i}\right)-{\pi }_{\widetilde{B}}^{2}\left({x}_{i}\right)\right)}^{2}}.$$

## The differential measure

Recently, Song et al. [52] pointed out the difference between classical distance measures and psychological distance measure. The psychological distance can overcome the counter-intuitive phenomenon of the classical distance measures that do not integrate the background information of alternatives and neglect their competitive relationships. For example, when we rank the three alternatives $A, B,$ and $C$ with the Pythagorean fuzzy evaluations $\widetilde{A}=\left(0.8, 0.2\right), \widetilde{B}=\left(0.7, 0.3\right), \; \mathrm{and} \, \widetilde{C}=\left(0.7, 0.1\right)$ using the score function (10), alternative $A$ is the best option. Comparing the evaluations of $B$ and $C$, it is clear that $C$ is better than $B$ having an equal degree of support but less degree of opposition. When using distance measures, either the normalized Hamming distance (13) or the normalized Euclidean distance (14), we get $d\left(\widetilde{A},\widetilde{C}\right)>d\left(\widetilde{A},\widetilde{B}\right)$. This indicates that $\widetilde{B}$ is closer to $\widetilde{A}$ than $\widetilde{C}$, i.e., $B$ is a better substitute for $A$ than $C$. Hence, we cannot count on the conventional distance measures to reflect the actual preference of different alternatives. For counter-examples that exemplify the defects of the extant distance measures, the reader is referred to Xiao and Ding [48] and Huang et al. [50].

Sure, $\widetilde{A}$ is better than $\widetilde{B}$, since it has a higher degree of support and a lower degree of opposition. Therefore, we can say that “$\widetilde{A}$ is superior to $\widetilde{B}$” or “$\widetilde{B}$ is inferior to $\widetilde{A}$”. Similarly, when comparing the alternatives $B$ and $C$, it is clear that $C$ is better than $B$ having equal support and lower opposition.

When comparing the alternatives $A$ and $C$, although $\widetilde{A}$ is better than $\widetilde{C}$ regarding the support, $\widetilde{C}$ is better than $\widetilde{A}$ regarding the opposition. In the conventional distance measures, an increase in the membership degree is equally treated as an increase in the non-membership degree, although each parameter has a different implication. Therefore, signed distance should be used to count for support and opposition. A step in the membership direction is considered a positive step, while a step in the non-membership direction will be considered a negative step. Therefore, the differential measure is expressed using a PFS $\left({\mu }_{\widetilde{D}},{\upsilon }_{\widetilde{D}}\right)$ to account for the two parameters. The support ${\mu }_{\widetilde{D}}$ increases with the increase in the difference between membership degrees, and the opposition ${\upsilon }_{\widetilde{D}}$ increases with the increase in the difference between non-membership degrees and vice versa.

Consider the alternatives $A$ and $B$ with the Pythagorean fuzzy evaluations $\widetilde{A}=\left(0.6, 0.6\right),$ and $\widetilde{B}=\left(0.4, 0.4\right)$. Applying the score function (10) and the accuracy function (11), we get

$${Sc}_{ZX}\left(\widetilde{A}\right)={Sc}_{ZX}\left(\widetilde{B}\right)=0, {\mathrm{Ac}}_{PY}\left(\widetilde{A}\right)=0.72, \; \mathrm{ and } \; {\mathrm{Ac}}_{PY}\left(\widetilde{B}\right)=0.32.$$

The parameters of a PFS $\left(\mu ,\upsilon \right)$ have a physical interpretation as “vote for” and “vote against”, respectively [79]. Hence, for alternative $A$, the vote for resolution is 6 in favor, and 6 against. Meanwhile, for alternative $B$, the vote for resolution is 4 in favor, and 4 against. In both evaluations, a tie occurs and the Pythagorean fuzzy evaluations $\widetilde{A}$ and $\widetilde{B}$ have the same impact. In this case, the accuracy function (11) which accounts for the amount of information is biased to $\widetilde{A}$. Of course, $\widetilde{A}$ conveys more information than $\widetilde{B}$. Yet, in such a case, the excess of information obtained by the PFS is relevant, since it only makes one option look better. There is no compelling reason for selecting one option over another [82]. This brings us to the basic notion of a score function, better PFSs are those with high values of $\mu$ and small values of $\upsilon$. What matters is simply the difference between the support and the opposition.

### Rules of differentiation

Since the Pythagorean fuzzy human assessment encompasses two conflicting parameters, it is more convenient to express the differential measure by a PFS $\left({\mu }_{\widetilde{D}},{\upsilon }_{\widetilde{D}}\right)$. For the two PFSs $\widetilde{A}=\left({\mu }_{\widetilde{A}},{\upsilon }_{\widetilde{A}}\right) \; \mathrm{and} \; \widetilde{B}=\left({\mu }_{\widetilde{B}},{\upsilon }_{\widetilde{B}}\right)$, if ${\mu }_{\widetilde{A}}^{2}{-\mu }_{\widetilde{B}}^{2}={\upsilon }_{\widetilde{A}}^{2}{-\upsilon }_{\widetilde{B}}^{2}$, then ${\mu }_{\widetilde{D}}= {\upsilon }_{\widetilde{D}}=0.5$ indicating equal preference. If ${\mu }_{\widetilde{A}}^{2}{-\mu }_{\widetilde{B}}^{2}>{\upsilon }_{\widetilde{A}}^{2}{-\upsilon }_{\widetilde{B}}^{2}$, then ${\mu }_{\widetilde{D}}$ increases and ${\upsilon }_{\widetilde{D}}$ decreases. The increase in the difference between the support and the opposition indicates more preference for $\widetilde{A}$. Else if ${\mu }_{\widetilde{A}}^{2}{-\mu }_{\widetilde{B}}^{2}<{\upsilon }_{\widetilde{A}}^{2}{-\upsilon }_{\widetilde{B}}^{2}$, then ${\mu }_{\widetilde{D}}$ decreases and ${\upsilon }_{\widetilde{D}}$ increases. The increase in this difference indicates less preference for $\widetilde{A}$. The score of the differential measure represents the degree of superiority or inferiority.

The differential measure between two PFSs $\widetilde{A}=\left({\mu }_{\widetilde{A}},{\upsilon }_{\widetilde{A}}\right), \; \mathrm{and} \; \widetilde{B}=\left({\mu }_{\widetilde{B}},{\upsilon }_{\widetilde{B}}\right)$ is calculated as follows. The datum for the membership and non-membership degrees is one. If the membership degree of $\widetilde{A}$ is greater than $\widetilde{B}$, the difference between the grades is added to the one indicating a positive step; if the membership degree of $\widetilde{A}$ is less than $\widetilde{B}$, the difference between the grades is subtracted from the one indicating a negative step. The non-membership degree is treated similarly. Then, the ordered pair is normalized. Accordingly, the differential measure can be defined as follows.

#### Definition 9

A differential measure between two Pythagorean fuzzy sets (PFSs) is a preference relation between two PFSs based on the closeness of their membership and non-membership degrees according to their position in the attribute space. The differential measure between two PFSs $\widetilde{A}=\left({\mu }_{\widetilde{A}},{\upsilon }_{\widetilde{A}}\right), \; \mathrm{and} \; \widetilde{B}=\left({\mu }_{\widetilde{B}},{\upsilon }_{\widetilde{B}}\right)$ is given by

15 $$\begin{aligned} Diff\left(\widetilde{A},\widetilde{B}\right) & =\left({\mu }_{\widetilde{D}},{\upsilon }_{\widetilde{D}}\right)=\left(\frac{1+\left({\mu }_{\widetilde{A}}^{2}-\mu _{\widetilde{B}}^{2}\right)}{2+\left({\mu }_{\widetilde{A}}^{2}+{\upsilon }_{\widetilde{A}}^{2}\right)-\left({\mu }_{\widetilde{B}}^{2}+{\upsilon }_{\widetilde{B}}^{2}\right)},\right. \\ & \quad \left.  \frac{1+\left({\upsilon }_{\widetilde{A}}^{2}-{\upsilon }_{\widetilde{B}}^{2}\right)}{2+\left({\mu }_{\widetilde{A}}^{2}+{\upsilon }_{\widetilde{A}}^{2}\right)-\left({\mu }_{\widetilde{B}}^{2}+{\upsilon }_{\widetilde{B}}^{2}\right)}\right).\end{aligned}$$

#### Definition 10.

Two PFSs $\widetilde{A}=\left({\mu }_{\widetilde{A}},{\upsilon }_{\widetilde{A}}\right), \; \mathrm{and} \; \widetilde{B}=\left({\mu }_{\widetilde{B}},{\upsilon }_{\widetilde{B}}\right)$ can be classified using the differential measure (15) as follows:

1. If $\mathrm{Diff}\left(\widetilde{A},\widetilde{B}\right)=\left({0.5,0.5}\right),$ then $\widetilde{A}$ and $\widetilde{B}$ are equivalent $\widetilde{A}\cong \widetilde{B}$ with a degree of superiority $\mathrm{DoS}\left(\widetilde{A},\widetilde{B}\right)=\mathrm{DoS}\left(\widetilde{B},\widetilde{A}\right)=0 ,$ and a degree of inferiority $\mathrm{DoN}\left(\widetilde{A},\widetilde{B}\right)=\mathrm{DoN}\left(\widetilde{B},\widetilde{A}\right)=0$.
  If ${\mu }_{\widetilde{A}}={\mu }_{\widetilde{B}}$ and ${\upsilon }_{\widetilde{A}}={\upsilon }_{\widetilde{B}}$, then $\widetilde{A}$ and $\widetilde{B}$ are identical $\widetilde{A}\equiv \widetilde{B}$.
2. If $\mathrm{Diff}\left(\widetilde{A},\widetilde{B}\right)=\left({\mu }_{\widetilde{D}},{\upsilon }_{\widetilde{D}}\right)$ with ${\mu }_{\widetilde{D}}>0.5$, then $\widetilde{A}$ is superior to $\widetilde{B}$
  $\widetilde{A}\succ \widetilde{B}$ with a degree of superiority $\mathrm{DoS}\left(\widetilde{A},\widetilde{B}\right)={\mu }_{\widetilde{D}}^{2}\left(x\right)-{\upsilon }_{\widetilde{D}}^{2}\left(x\right)>0$.
3. If $\mathrm{Diff}\left(\widetilde{A},\widetilde{B}\right)=\left({\mu }_{\widetilde{D}},{\upsilon }_{\widetilde{D}}\right)$ with ${\mu }_{\widetilde{D}}<0.5$, then $\widetilde{A}$ is inferior to $\widetilde{B}$

$\widetilde{A}\prec \widetilde{B}$ with a degree of inferiority $\mathrm{DoN}\left(\widetilde{A},\widetilde{B}\right)={\mu }_{\widetilde{D}}^{2}\left(x\right)-{\upsilon }_{\widetilde{D}}^{2}\left(x\right)<0$.

#### Proposition 1

*For the PFSs* $\widetilde{A}=\left({\mu }_{\widetilde{A}},{\upsilon }_{\widetilde{A}}\right) , \widetilde{B}=\left({\mu }_{\widetilde{B}},{\upsilon }_{\widetilde{B}}\right), \; \mathrm{and} \; \widetilde{C}=\left({\mu }_{\widetilde{c}},{\upsilon }_{\widetilde{c}}\right)$

(i) *if* $\widetilde{A}\succ \widetilde{B}$ *and* $\widetilde{B}\succ \widetilde{C}$ , * then* $\widetilde{A}\succ \widetilde{C}$.
(ii) *if* $\widetilde{A}\prec \widetilde{B}$ *and* $\widetilde{B}\prec \widetilde{C}$, * then* $\widetilde{A}\prec \widetilde{C}$.

#### Proof

The proof follows from the definition of the score function (10).

If $\widetilde{A}\succ \widetilde{B}\Rightarrow {\mu }_{\widetilde{A}}^{2}{-\mu }_{\widetilde{B}}^{2}>{\upsilon }_{\widetilde{A}}^{2}{-\upsilon }_{\widetilde{B}}^{2}$ from (15), hence ${\mu }_{\widetilde{A}}^{2}{-\upsilon }_{\widetilde{A}}^{2}>{\mu }_{\widetilde{B}}^{2}{-\upsilon }_{\widetilde{B}}^{2}$

If $$\widetilde{B}\succ \widetilde{C}\Rightarrow {\mu }_{\widetilde{B}}^{2}{-\upsilon }_{\widetilde{B}}^{2}>{\mu }_{\widetilde{C}}^{2}{-\upsilon }_{\widetilde{C}}^{2}$$.

Then, $${\mu }_{\widetilde{A}}^{2}{-\upsilon }_{\widetilde{A}}^{2}>{\mu }_{\widetilde{C}}^{2}{-\upsilon }_{\widetilde{C}}^{2}\Rightarrow {\mu }_{\widetilde{A}}^{2}{-\mu }_{\widetilde{C}}^{2}>{\upsilon }_{\widetilde{A}}^{2}{-\upsilon }_{\widetilde{C}}^{2}$$ and $$\widetilde{A}\succ \widetilde{C}$$.

If $\widetilde{A}\prec \widetilde{B}\Rightarrow {\mu }_{\widetilde{A}}^{2}{-\mu }_{\widetilde{B}}^{2}<{\upsilon }_{\widetilde{A}}^{2}{-\upsilon }_{\widetilde{B}}^{2}$ from (15), hence ${\mu }_{\widetilde{A}}^{2}{-\upsilon }_{\widetilde{A}}^{2}<{\mu }_{\widetilde{B}}^{2}{-\upsilon }_{\widetilde{B}}^{2}$.

If $$\widetilde{B}\succ \widetilde{C}\Rightarrow {\mu }_{\widetilde{B}}^{2}{-\upsilon }_{\widetilde{B}}^{2}<{\mu }_{\widetilde{C}}^{2}{-\upsilon }_{\widetilde{C}}^{2}$$.

Then, $$\Rightarrow {\mu }_{\widetilde{A}}^{2}{-\upsilon }_{\widetilde{A}}^{2}<{\mu }_{\widetilde{C}}^{2}{-\upsilon }_{\widetilde{C}}^{2}$$ and $$\widetilde{A}\prec \widetilde{C}$$.

#### Proposition 2

*For the PFSs* $\widetilde{A}=\left({\mu }_{\widetilde{A}},{\upsilon }_{\widetilde{A}}\right) \mathrm{and }\; {\widetilde{A}}^{c}=\left({\upsilon }_{\widetilde{A}},{\mu }_{\widetilde{A}}\right)$.

$$\mathrm{Diff}\left(\widetilde{A},{\widetilde{A}}^{c}\right)=\mathrm{Diff}^{c}\left({\widetilde{A}}^{c},\widetilde{A}\right),$$

And if $\widetilde{A}$ is superior to ${\widetilde{A}}^{c}$, then

$\mathrm{DoN}\left({\widetilde{A}}^{c},\widetilde{A}\right)= -\mathrm{DoS}\left(\widetilde{A},{\widetilde{A}}^{c}\right)$, and vice versa.

#### ***Proof***

$$ \begin{aligned} {\text{Diff}}\left( {\tilde{A},\tilde{A}^{c} } \right) & = \left( {\mu_{{\tilde{D}}} ,\upsilon_{{\tilde{D}}} } \right) = \left(\frac{{1 + \left( {\mu_{{\tilde{A}}}^{2} - \upsilon_{{\tilde{A}}}^{2} } \right)}}{{2 + \left( {\mu_{{\tilde{A}}}^{2} + \upsilon_{{\tilde{A}}}^{2} } \right)- \left( {\mu_{{\tilde{A}}}^{2} + \upsilon_{{\tilde{A}}}^{2} } \right)}},\right.\\ &\quad \left. \frac{{1 + \left( {\upsilon_{{\tilde{A}}}^{2} - \mu_{{\tilde{A}}}^{2} } \right)}}{{2 + \left( {\mu_{{\tilde{A}}}^{2} + \upsilon_{{\tilde{A}}}^{2} } \right) - \left( {\mu_{{\tilde{A}}}^{2} + \upsilon_{{\tilde{A}}}^{2} } \right)}} \right) \\ & = \left( {\frac{{1 + \left( {\mu_{{\tilde{A}}}^{2} - \upsilon_{{\tilde{A}}}^{2} } \right)}}{2},\frac{{1 + \left( {\upsilon_{{\tilde{A}}}^{2} - \mu_{{\tilde{A}}}^{2} } \right)}}{2}} \right), \\ \end{aligned} $$

$$ \begin{aligned} &{\text{Diff}}\left( {\tilde{A}^{c} ,\tilde{A}} \right)\\ &\quad = \left( {\frac{{1 + \left( {\upsilon_{{\tilde{A}}}^{2} - \mu_{{\tilde{A}}}^{2} } \right)}}{{2 + \left( {\mu_{{\tilde{A}}}^{2} + \upsilon_{{\tilde{A}}}^{2} } \right) - \left( {\mu_{{\tilde{A}}}^{2} + \upsilon_{{\tilde{A}}}^{2} } \right)}},\frac{{1 + \left( {\mu_{{\tilde{A}}}^{2} - \upsilon_{{\tilde{A}}}^{2} } \right)}}{{2 + \left( {\mu_{{\tilde{A}}}^{2} + \upsilon_{{\tilde{A}}}^{2} } \right) - \left( {\mu_{{\tilde{A}}}^{2} + \upsilon_{{\tilde{A}}}^{2} } \right)}}} \right) \\ & = \left( {\frac{{1 + \left( {\upsilon_{{\tilde{A}}}^{2} - \mu_{{\tilde{A}}}^{2} } \right)}}{2},\frac{{1 + \left( {\mu_{{\tilde{A}}}^{2} - \upsilon_{{\tilde{A}}}^{2} } \right)}}{2}} \right) = \left( {\upsilon_{{\tilde{D}}} ,\mu_{{\tilde{D}}} } \right). \\ \end{aligned} $$

### Numerical examples

In this section, some examples are presented to illustrate the application of differential measures.

#### Example 1.

Let $$\widetilde{A}=\left(\mathrm{0.9,0.1}\right) \; \mathrm{and} \; \widetilde{B}=\left(\mathrm{0.8,0.2}\right)$$

$$\begin{aligned}\mathrm{Diff}\left(\widetilde{A},\widetilde{B}\right)& =\left(\frac{1+\left({0.9}^{2}-{0.8}^{2}\right)}{2+\left({0.9}^{2}+{0.1}^{2}\right)-\left({0.8}^{2}+{0.2}^{2}\right)},\right.\\ &  \left. \quad\frac{1+\left({0.1}^{2}-{0.2}^{2}\right)}{2+\left({0.9}^{2}+{0.1}^{2}\right)-\left({0.8}^{2}+{0.2}^{2}\right)}\right)\\& \quad =\left({0.55,0.45}\right),\end{aligned}$$

$$ \begin{aligned}\mathrm{Diff}\left(\widetilde{B},\widetilde{A}\right)& =\left(\frac{1+\left({0.8}^{2}-{0.9}^{2}\right)}{2+\left({0.8}^{2}+{0.2}^{2}\right)-\left({0.9}^{2}+{0.1}^{2}\right)},\right.\\ & \quad\left. \frac{1+\left({0.2}^{2}-{0.1}^{2}\right)}{2+\left({0.8}^{2}+{0.2}^{2}\right)-\left({0.9}^{2}+{0.1}^{2}\right)}\right)\\ & =\left(\mathrm{0.45,0.55}\right).\end{aligned}$$

Then, $\widetilde{A}$ is superior to $\widetilde{B}$ with a degree of superiority $\mathrm{DoS}\left(\widetilde{A},\widetilde{B}\right)=0.1$, or $\widetilde{B}$ is inferior to $\widetilde{A}$ with a degree of inferiority $\mathrm{DoN}\left(\widetilde{B},\widetilde{A}\right)=-0.1.$

#### Example 2.

Let $$\widetilde{A}=\left(\mathrm{0.9,0.1}\right) \; \mathrm{and} \; \widetilde{B}=\left(\mathrm{0.8,0.3}\right)$$

$$ \begin{aligned}\mathrm{Diff}\left(\widetilde{A},\widetilde{B}\right)&=\left(\frac{1+\left({0.9}^{2}-{0.8}^{2}\right)}{2+\left({0.9}^{2}+{0.1}^{2}\right)-\left({0.8}^{2}+{0.3}^{2}\right)},\right.\\ &\left. \quad\frac{1+\left({0.1}^{2}-{0.3}^{2}\right)}{2+\left({0.9}^{2}+{0.1}^{2}\right)-\left({0.8}^{2}+{0.3}^{2}\right)}\right)\\ & =\left(\mathrm{0.56,0.44}\right)\end{aligned}$$

$$\begin{aligned}\mathrm{Diff}\left(\widetilde{B},\widetilde{A}\right)&=\left(\frac{1+\left({0.8}^{2}-{0.9}^{2}\right)}{2+\left({0.8}^{2}+{0.3}^{2}\right)-\left({0.9}^{2}+{0.1}^{2}\right)},\right.\\ &\left. \quad\frac{1+\left({0.3}^{2}-{0.1}^{2}\right)}{2+\left({0.8}^{2}+{0.3}^{2}\right)-\left({0.9}^{2}+{0.1}^{2}\right)}\right)\\ &=\left(\mathrm{0.43,0.57}\right).\end{aligned}$$

Then, $\widetilde{A}$ is superior to $\widetilde{B}$ with a degree of superiority $\mathrm{DoS}\left(\widetilde{A},\widetilde{B}\right)=0.12$, or $\widetilde{B}$ is inferior to $\widetilde{A}$ with a degree of inferiority $\mathrm{DoN}\left(\widetilde{B},\widetilde{A}\right)=-0.14.$

It is clear that the degree of superiority of $\widetilde{A}$ and the degree of inferiority of $\widetilde{B}$, in this example, increased with the increase of the opposition of $\widetilde{B}$ than in the previous example.

#### Example 3.

Let $$\widetilde{A}=\left(\mathrm{0.8,0.2}\right) \; \mathrm{and} \; \widetilde{B}=\left(\mathrm{0.7,0.1}\right)$$

$$\begin{aligned}\mathrm{Diff}\left(\widetilde{A},\widetilde{B}\right)&=\left(\frac{1+\left({0.8}^{2}-{0.7}^{2}\right)}{2+\left({0.8}^{2}+{0.2}^{2}\right)-\left({0.7}^{2}+{0.1}^{2}\right)},\right.\\ & \quad\left.\frac{1+\left({0.2}^{2}-{0.1}^{2}\right)}{2+\left({0.8}^{2}+{0.2}^{2}\right)-\left({0.7}^{2}+{0.1}^{2}\right)}\right)\\ & =\left(\mathrm{0.53,0.47}\right)\end{aligned}$$

$$\begin{aligned}\mathrm{Diff}\left(\widetilde{B},\widetilde{A}\right)&=\left(\frac{1+\left({0.7}^{2}-{0.8}^{2}\right)}{2+\left({0.7}^{2}+{0.1}^{2}\right)-\left({0.8}^{2}+{0.2}^{2}\right)},\right.\\ &\quad\left.\frac{1+\left({0.1}^{2}-{0.2}^{2}\right)}{2+\left({0.8}^{2}+{0.2}^{2}\right)-\left({0.8}^{2}+{0.2}^{2}\right)}\right)\\ &=\left(\mathrm{0.47,0.53}\right).\end{aligned}$$

Then, in this case, $\widetilde{A}\succ \widetilde{B}$ with $\mathrm{DoS}\left(\widetilde{A},\widetilde{B}\right)=0.06$, $\widetilde{B}\prec \widetilde{A}$ with $\mathrm{DoN}\left(\widetilde{B},\widetilde{A}\right)=-0.06.$

#### Example 4.

Let $$\widetilde{A}=\left(\mathrm{0.6,0.6}\right) \; \mathrm{and} \; \widetilde{B}=\left(\mathrm{0.3,0.3}\right)$$

$$\begin{aligned}\mathrm{Diff}\left(\widetilde{A},\widetilde{B}\right)&=\left(\frac{1+\left({0.6}^{2}-{0.3}^{2}\right)}{2+\left({0.6}^{2}+{0.6}^{2}\right)-\left({0.3}^{2}+{0.3}^{2}\right)},\right.\\ &\quad\left.\frac{1+\left({0.6}^{2}-{0.3}^{2}\right)}{2+\left({0.6}^{2}+{0.6}^{2}\right)-\left({0.3}^{2}+{0.3}^{2}\right)}\right)\\ & =\left(0.5,0.5\right)\end{aligned}$$

$$\begin{aligned}\mathrm{Diff}\left(\widetilde{B},\widetilde{A}\right)&=\left(\frac{1+\left({0.3}^{2}-{0.6}^{2}\right)}{2+\left({0.3}^{2}+{0.3}^{2}\right)-\left({0.6}^{2}+{0.6}^{2}\right)},\right.\\ & \left.\quad \frac{1+\left({0.3}^{2}-{0.6}^{2}\right)}{2+\left({0.3}^{2}+{0.3}^{2}\right)-\left({0.6}^{2}+{0.6}^{2}\right)}\right)\\ & =\left(\mathrm{0.5,0.5}\right).\end{aligned}$$

Here, $\widetilde{A}$ is equivalent to $\widetilde{B}$, since they have the same difference between support and opposition. This equivalence can be justified using the similarity measure proposed by Zhang [58]

16 $$\begin{aligned}\mathrm{Sim}\left(\widetilde{A},\widetilde{P}\right)&=\frac{d\left(\widetilde{A},{\widetilde{P}}^{c}\right)}{d\left(\widetilde{A},\widetilde{P}\right)+d\left(\widetilde{A},{\widetilde{P}}^{c}\right)}.\end{aligned}$$

Suppose $\widetilde{P}$ is the positive ideal solution $\left(\mathrm{1,0}\right)$, and its complement ${\widetilde{P}}^{c}$ is the negative ideal solution $\left(\mathrm{0,1}\right)$. Using the normalized Euclidean distance (14)

$${d}_{\mathrm{NE}}\left(\widetilde{A},\widetilde{P}\right)=0.5557 \; \mathrm{and} \; {d}_{\mathrm{NE}}\left(\widetilde{B},\widetilde{P}\right)=0.8685,$$

$${d}_{\mathrm{NE}}\left(\widetilde{A},{\widetilde{P}}^{c}\right)=0.5557 \; \mathrm{and} \; {d}_{\mathrm{NE}}\left(\widetilde{B},{\widetilde{P}}^{c}\right)=0.8685.$$

Although $\widetilde{A}$ is closer to $\widetilde{P}$ than $\widetilde{B}$, it is also closer to ${\widetilde{P}}^{c}$ than $\widetilde{B}.$

Calculating the degrees of similarity

$$\mathrm{Sim}\left(\widetilde{A},\widetilde{P}\right)=\frac{{d}_{E}\left(\widetilde{A},{\widetilde{P}}^{c}\right)}{{d}_{E}\left(\widetilde{A},\widetilde{P}\right)+{d}_{E}\left(\widetilde{A},{\widetilde{P}}^{c}\right)}=\frac{0.5557}{0.5557+0.5557 }=0.5,$$

$$\mathrm{Sim}\left(\widetilde{B},\widetilde{P}\right)=\frac{{d}_{E}\left(\widetilde{B},{\widetilde{P}}^{c}\right)}{{d}_{E}\left(\widetilde{B},\widetilde{P}\right)+{d}_{E}\left(B,{\widetilde{P}}^{c}\right)}=\frac{0.8685 }{0.8685+0.8685 }=0.5.$$

Both points have the same degree of similarity to the positive ideal solution. Then, both have the same effect when making a decision. Although the accuracy function (11) of $\widetilde{A}$ is greater than that of $\widetilde{B}$, i.e., $\widetilde{A}$ has more information than $\widetilde{B}$, the extra information provided by $\widetilde{A}$ is non-instrumental. Additional information is used only with the need to determine the preference to decide and to facilitate the choice [82]. By intuition, we cannot recommend one of these alternatives. In both PFSs, the percentage of support is equal to the percentage of opposition; simply, they have the same effect.

#### Example 5.

Let $$\widetilde{A}=\left(\mathrm{0.5,0.1}\right) \; \mathrm{and} \; {\widetilde{A}}^{c}=\left(\mathrm{0.1,0.5}\right)$$

$$\mathrm{Diff}\left(\widetilde{A},{\widetilde{A}}^{c}\right)=\left(\frac{1+\left({0.5}^{2}-{0.1}^{2}\right)}{2+\left({0.5}^{2}+{0.1}^{2}\right)-\left({0.1}^{2}+{0.5}^{2}\right)},\frac{1+\left({0.1}^{2}-{0.5}^{2}\right)}{2+\left({0.5}^{2}+{0.1}^{2}\right)-\left({0.1}^{2}+{0.5}^{2}\right)}\right)=\left(\mathrm{0.62,0.38}\right),$$

$$\mathrm{Diff}\left({\widetilde{A}}^{c},\widetilde{A}\right)=\left(\frac{1+\left({0.1}^{2}-{0.5}^{2}\right)}{2+\left({0.1}^{2}+{0.5}^{2}\right)-\left({0.5}^{2}+{0.1}^{2}\right)},\frac{1+\left({0.5}^{2}-{0.1}^{2}\right)}{2+\left({0.1}^{2}+{0.5}^{2}\right)-\left({0.5}^{2}+{0.1}^{2}\right)}\right)=\left(\mathrm{0.38,0.62}\right),$$

$$\mathrm{DoS}\left(\widetilde{A},{\widetilde{A}}^{c}\right)=0.24 \; \mathrm{and} \; \mathrm{DoN}\left({\widetilde{A}}^{c},\widetilde{A}\right)=-0.24.$$

#### Example 6.

Let $$\widetilde{A}=\left(\mathrm{0.9,0.1}\right), \widetilde{B}=\left(\mathrm{0.8,0.2}\right), \; \mathrm{and} \; \widetilde{C}=\left(\mathrm{0.7,0.3}\right)$$

$$\mathrm{Diff}\left(\widetilde{A},\widetilde{B}\right)=\left(0.54,0.45\right), \mathrm{DoS}\left(\widetilde{A},\widetilde{B}\right)=0.1,\mathrm{Diff}\left(\widetilde{B},\widetilde{A}\right)=\left(0.45,0.55\right), \mathrm{DoN}\left(\widetilde{B},\widetilde{A}\right)=-0.1.$$

$$\mathrm{Diff}\left(\widetilde{B},\widetilde{C}\right)=\left(0.55,0.45\right),\mathrm{DoS}\left(\widetilde{B},\widetilde{C}\right)=0.1,\mathrm{Diff}\left(\widetilde{C},\widetilde{B}\right)=\left(0.45,0.55\right), \mathrm{DoN}\left(\widetilde{C},\widetilde{B}\right)=-0.1,$$

$$\mathrm{Diff}\left(\widetilde{A},\widetilde{C}\right)=\left(0.59,0.41\right),\mathrm{DoS}\left(\widetilde{A},\widetilde{C}\right)=0.2,\mathrm{ Diff}\left(\widetilde{C},\widetilde{A}\right)=\left(0.40,0.60\right),\mathrm{DoN}\left(\widetilde{C},\widetilde{A}\right)=-0.18.$$

Hence, $\widetilde{A}\succ \widetilde{B}$ and $\widetilde{B}\succ \widetilde{C}$, we get $\widetilde{A}\succ \widetilde{C}$.

### Comparison between the differential measure and some information measures

This subsection aims to illustrate how DFM works versus how some extant information measures work. Hence, the proposed DFM is compared with some extant information measures to demonstrate the difference in the results and the reasonability of the DFM.

The DFM between two PFSs $\widetilde{\mathbf{A}}=\left\{{\widetilde{A}}_{1},{\widetilde{A}}_{2},\dots ,{\widetilde{A}}_{n}\right\}$ and $\widetilde{\mathbf{B}}=\left\{{\widetilde{B}}_{1},{\widetilde{B}}_{2},\dots ,{\widetilde{B}}_{n}\right\}$ is defined as

17 $$\mathrm{Diff}\left(\widetilde{\mathbf{A}},\widetilde{\mathbf{B}}\right)={\mathrm{PFWA}}_{Y}\left\{\mathrm{Diff}\left({\widetilde{A}}_{i},{\widetilde{B}}_{i}\right)\right\}, {w}_{i}=\frac{1}{n}.$$

Three distance measures are employed in comparison, the normalized Hamming distance (13), the normalized Euclidean distance (14), and the Pythagorean fuzzy Jensen–Shannon (PFSJS) distance which is defined as follows [48]:

18 $${d}_{\mathrm{PFSJS}}\left(\widetilde{\mathbf{A}},\widetilde{\mathbf{B}}\right)=\frac{1}{n}\sum_{i=1}^{n}\sqrt{\frac{1}{2}\left[\begin{array}{c}\left({\mu }_{{\widetilde{A}}_{i}}^{2}\mathrm{log}\frac{2{\mu }_{{\widetilde{A}}_{i}}^{2}}{{\mu }_{{\widetilde{A}}_{i}}^{2}+{\mu }_{{\widetilde{B}}_{i}}^{2}}+{\upsilon }_{{\widetilde{A}}_{i}}^{2}\mathrm{log}\frac{2{\upsilon }_{{\widetilde{A}}_{i}}^{2}}{{\upsilon }_{{\widetilde{A}}_{i}}^{2}+{\upsilon }_{{\widetilde{B}}_{i}}^{2}}+{\pi }_{{\widetilde{A}}_{i}}^{2}\mathrm{log}\frac{2{\pi }_{{\widetilde{A}}_{i}}^{2}}{{\pi }_{{\widetilde{A}}_{i}}^{2}+{\pi }_{{\widetilde{B}}_{i}}^{2}}\right)+\\ \left({\mu }_{{\widetilde{B}}_{i}}^{2}\mathrm{log}\frac{2{\mu }_{{\widetilde{B}}_{i}}^{2}}{{\mu }_{{\widetilde{A}}_{i}}^{2}+{\mu }_{{\widetilde{B}}_{i}}^{2}}+{\upsilon }_{{\widetilde{B}}_{i}}^{2}\mathrm{log}\frac{2{\upsilon }_{{\widetilde{B}}_{i}}^{2}}{{\upsilon }_{{\widetilde{A}}_{i}}^{2}+{\upsilon }_{{\widetilde{B}}_{i}}^{2}}+{\pi }_{{\widetilde{B}}_{i}}^{2}\mathrm{log}\frac{2{\pi }_{{\widetilde{B}}_{i}}^{2}}{{\pi }_{{\widetilde{A}}_{i}}^{2}+{\pi }_{{\widetilde{B}}_{i}}^{2}}\right)\end{array}\right],}$$

where ${\pi }_{i}$ is defined as given in (3).

The most commonly used similarity measures are distance-based similarity measures, and vector-based similarity measures, for instance, Cosine, Dice, and Jaccard similarity measures. Distance-based similarity measures utilize different distance measures, i.e., distance and similarity measure is a dual concept. A general form of distance-based similarity measure between two PFSs can be defined as

19 $${\mathrm{Sim}}_{db}\left(\widetilde{\mathbf{A}},\widetilde{\mathbf{B}}\right)=\frac{f\left(d\left(\widetilde{\mathbf{A}},\widetilde{\mathbf{B}}\right)\right)-f\left(1\right)}{f\left(0\right)-f\left(1\right)},$$

where $f$ is a monotonically decreasing function. Different kinds of similarity measures can be obtained using an appropriate function. For example, the simple linear function $f\left(x\right)=1-x$, a simple rational function $f\left(x\right)=1/(1+x)$, and an exponential function.$f\left(x\right)={e}^{-x}$ [63]. Since the main aim of the section is to clarify the difference between similarity measures and DFM, the simple linear function only is used

20 $${\mathrm{Sim}}_{db}\left(\widetilde{\mathbf{A}},\widetilde{\mathbf{B}}\right)=1-d\left(\widetilde{\mathbf{A}},\widetilde{\mathbf{B}}\right).$$

Additionally, the previously mentioned three vector-based similarity measures are employed in comparison. The cosine similarity measure is given by [57]

21 $${\mathrm{Sim}}_{\mathrm{cos}}\left(\widetilde{\mathbf{A}},\widetilde{\mathbf{B}}\right)=\frac{1}{n}\sum_{i=1}^{n}\frac{{\mu }_{{\widetilde{A}}_{i}}^{2}.{\mu }_{{\widetilde{B}}_{i}}^{2}+{\upsilon }_{{\widetilde{A}}_{i}}^{2}.{\upsilon }_{{\widetilde{B}}_{i}}^{2}}{\sqrt{{\mu }_{{\widetilde{A}}_{i}}^{4}+{\upsilon }_{{\widetilde{A}}_{i}}^{4}}\sqrt{{\mu }_{{\widetilde{B}}_{i}}^{4}+{\upsilon }_{{\widetilde{B}}_{i}}^{4}}}.$$

The Dice similarity measure is defined by [49]

22 $${\mathrm{Sim}}_{\mathrm{Dice}}\left(\widetilde{\mathbf{A}},\widetilde{\mathbf{B}}\right)=\frac{1}{n}\sum_{i=1}^{n}\frac{2\left({\mu }_{{\widetilde{A}}_{i}}^{2}.{\mu }_{{\widetilde{B}}_{i}}^{2}+{\upsilon }_{{\widetilde{A}}_{i}}^{2}.{\upsilon }_{{\widetilde{B}}_{i}}^{2}\right)}{\left(\left({\mu }_{{\widetilde{A}}_{i}}^{4}+{\upsilon }_{{\widetilde{A}}_{i}}^{4}\right)+\left({\mu }_{{\widetilde{B}}_{i}}^{4}+{\upsilon }_{{\widetilde{B}}_{i}}^{4}\right)\right)}.$$

The Jaccard similarity measure is defined by [50]

23 $${\mathrm{Sim}}_{\mathrm{Jaccard}}\left(\widetilde{\mathbf{A}},\widetilde{\mathbf{B}}\right)=\frac{1}{n}\sum_{i=1}^{n}\frac{\left({\mu }_{{\widetilde{A}}_{i}}^{2}.{\mu }_{{\widetilde{B}}_{i}}^{2}+{\upsilon }_{{\widetilde{A}}_{i}}^{2}.{\upsilon }_{{\widetilde{B}}_{i}}^{2}\right)}{\left(\left({\mu }_{{\widetilde{A}}_{i}}^{4}+{\upsilon }_{{\widetilde{A}}_{i}}^{4}\right)+\left({\mu }_{{\widetilde{B}}_{i}}^{4}+{\upsilon }_{{\widetilde{B}}_{i}}^{4}\right)-\left({\mu }_{{\widetilde{A}}_{i}}^{2}.{\mu }_{{\widetilde{B}}_{i}}^{2}+{\upsilon }_{{\widetilde{A}}_{i}}^{2}.{\upsilon }_{{\widetilde{B}}_{i}}^{2}\right)\right)}.$$

A correlation measure is employed in the comparison as well. Garg’s correlation measure defined by [73]

24 $${\mathrm{Corr}}_{\mathrm{Garg}}\left(\widetilde{\mathbf{A}},\widetilde{\mathbf{B}}\right)=\frac{\sum_{i=1}^{n}\left({\mu }_{{\widetilde{A}}_{i}}^{2}.{\mu }_{{\widetilde{B}}_{i}}^{2}+{\upsilon }_{{\widetilde{A}}_{i}}^{2}.{\upsilon }_{{\widetilde{B}}_{i}}^{2}+{\pi }_{{\widetilde{A}}_{i}}^{2}.{\pi }_{{\widetilde{B}}_{i}}^{2}\right)}{\sqrt{\sum_{i=1}^{n}\left({\mu }_{{\widetilde{A}}_{i}}^{4}+{\upsilon }_{{\widetilde{A}}_{i}}^{4}+{\pi }_{{\widetilde{A}}_{i}}^{4}\right)*\sum_{i=1}^{n}\left({\mu }_{{\widetilde{B}}_{i}}^{4}+{\upsilon }_{{\widetilde{B}}_{i}}^{4}+{\pi }_{{\widetilde{B}}_{i}}^{4}\right)}}.$$

It can be seen from the previous formulas that some information measures use the two independent degrees $\left(\mu ,\upsilon \right)$. Other measures use the three degrees $\left(\mu ,\upsilon ,\pi \right)$ to increase the discrimination capability of the measure, although the third degree depends on the two degrees $\left(\mu ,\upsilon \right)$.

Two PFSs ${\widetilde{\mathbf{B}}}_{1}$ and ${\widetilde{\mathbf{B}}}_{2}$ are compared to the PFSs $\widetilde{\mathbf{A}}$ using (17), (18), (20), (21), (22), (23), and (24). The PFSs are given as follows [48]:

$$\widetilde{\mathbf{A}}=\left\{{\widetilde{A}}_{1},{\widetilde{A}}_{2}\right\}=\left\{\left(\mathrm{0.55,0.45}\right),\left(\mathrm{0.63,0.55}\right)\right\},$$

$${\widetilde{\mathbf{B}}}_{1}=\left\{{\widetilde{B}}_{11},{\widetilde{B}}_{12}\right\}=\left\{\left(\mathrm{0.39,0.50}\right),\left(\mathrm{0.50,0.59}\right)\right\},\mathrm{and }\; {\widetilde{\mathbf{B}}}_{2}=\left\{\left(\mathrm{0.67,0.39}\right),\left(\mathrm{0.74,0.50}\right)\right\}.$$

It is clear by intuition that $\widetilde{\mathbf{A}}$ is better than ${\widetilde{\mathbf{B}}}_{1}$, since the degrees of membership are higher and the degrees of non-membership are lower. On the other hand, $\widetilde{\mathbf{A}}$ is worse than ${\widetilde{\mathbf{B}}}_{2}$ since the degrees of membership are lower and the degrees of non-membership are higher. The results of the comparison are given in Table 1.

**Table 1:** The comparison of some information measures

	$\left(\widetilde{\mathrm{A}},{\widetilde{\mathrm{B}}}_{1}\right)$			$\left(\widetilde{\mathrm{A}},{\widetilde{\mathrm{B}}}_{2}\right)$			
${d}_{\mathrm{NH}}$	0.15	${\mathrm{Sim}}_{\mathrm{dNH}}$	0.85	${d}_{\mathrm{NH}}$	0.15	${\mathrm{Sim}}_{\mathrm{dNH}}$	0.85
${d}_{\mathrm{NE}}$	0.1323	${\mathrm{Sim}}_{\mathrm{dNE}}$	0.8677	${d}_{\mathrm{NE}}$	0.1323	${\mathrm{Sim}}_{\mathrm{dNE}}$	0.8677
${d}_{\mathrm{PFSJS}}$	0.1463	${\mathrm{Sim}}_{\mathrm{dPFSJS}}$	0.8537	${d}_{\mathrm{PFSJS}}$	0.1323	${\mathrm{Sim}}_{\mathrm{dPFSJS}}$	0.8677
${\mathrm{Sim}}_{\mathrm{cos}}$	0.9318				0.9704		
${\mathrm{Sim}}_{\mathrm{Dice}}$	0.9155				0.9457		
${\mathrm{Sim}}_{\mathrm{Jaccard}}$	0.8456				0.8971		
${\mathrm{Corr}}_{\mathrm{Garg}}$	0.9550				0.9238		
DFM	$\left(\mathrm{0.5453,0.4548}\right)$ $\widetilde{\mathbf{A}}\succ {\widetilde{\mathbf{B}}}_{1}$ $\mathrm{DoS}\left(\widetilde{\mathbf{A}},{\widetilde{\mathbf{B}}}_{1}\right)=0.091$				$\left(\mathrm{0.4562,0.5438}\right)$ $\widetilde{\mathbf{A}}\prec {\widetilde{\mathbf{B}}}_{3}$ $\mathrm{DoN}\left(\widetilde{\mathbf{A}},{\widetilde{\mathbf{B}}}_{2}\right)=-0.088$		

From Table 1, ${\widetilde{\mathbf{B}}}_{1}$ and ${\widetilde{\mathbf{B}}}_{2}$ have the same distance from$\widetilde{\mathbf{A}}$**,** consequently the same degree of similarity to $\widetilde{\mathbf{A}}$ using the normalized Hamming (13) and the normalized Euclidean distance (14). The comparison also shows that ${\widetilde{\mathbf{B}}}_{2}$ is more similar to $\widetilde{\mathbf{A}}$ than ${\widetilde{\mathbf{B}}}_{1}$ using PFSJS distance measure, the cosine, Dice, and Jaccard similarity measures. Meanwhile, ${\widetilde{\mathbf{B}}}_{1}$ is more correlated with $\widetilde{\mathbf{A}}$ than ${\widetilde{\mathbf{B}}}_{2}$. None of these measures decided which PFS is better $\widetilde{\mathbf{A}}$ or ${\widetilde{\mathbf{B}}}_{1}$, $\widetilde{\mathbf{A}}$ or ${\widetilde{\mathbf{B}}}_{2}$, and to what extent. Moreover, if $\mathrm{Sim}\left(\widetilde{\mathbf{A}},{\widetilde{\mathbf{B}}}_{1}\right)=\mathrm{Sim}\left(\widetilde{\mathbf{A}},{\widetilde{\mathbf{B}}}_{2}\right)=0.85$, this does not necessarily imply that $\mathrm{Sim}\left({\widetilde{\mathbf{B}}}_{1},{\widetilde{\mathbf{B}}}_{2}\right)=1$. In this case, ${\mathrm{Sim}}_{\mathrm{Dice}}\left({\widetilde{\mathbf{B}}}_{1},{\widetilde{\mathbf{B}}}_{2}\right)=0.3763$. On the other hand, the proposed DFM can discriminate the PFSs. We have $\widetilde{\mathbf{A}}\boldsymbol{ }\succ {\widetilde{\mathbf{B}}}_{1}$, and $\widetilde{\mathbf{A}}\prec {\widetilde{\mathbf{B}}}_{2}$, from which we can conclude that ${\widetilde{\mathbf{B}}}_{1}\prec {\widetilde{\mathbf{B}}}_{2}$. By applying the DFM (17) directly, we get ${\widetilde{\mathbf{B}}}_{1}\prec {\widetilde{\mathbf{B}}}_{2}$ with $\mathrm{DoN}\left({\widetilde{\mathbf{B}}}_{1},{\widetilde{\mathbf{B}}}_{2}\right)=-0.1958$. The obtained result is close to that obtained using the normalized Hamming distance (13) and the normalized Euclidean distance (14) but more detailed. From the results, $\mathrm{DoS}\left(\widetilde{\mathbf{A}},{\widetilde{\mathbf{B}}}_{1}\right)\approx \left|\mathrm{DoN}\left(\widetilde{\mathbf{A}},{\widetilde{\mathbf{B}}}_{2}\right)\right|=0.09$, which means that ${\widetilde{\mathbf{B}}}_{1}$ and ${\widetilde{\mathbf{B}}}_{2}$ are similar in their difference from $\widetilde{\mathbf{A}}$**,** but in opposite directions, ${\widetilde{\mathbf{B}}}_{2}$ in the increment direction, while ${\widetilde{\mathbf{B}}}_{1}$ is in the decrement direction.

Regarding the computational complexity of the proposed DFM relative to the extant information measures, the number of operations required by the methods used in the comparison is calculated. For distance measures, the number of operations ranges from $(10n+2)$ for the normalized Hamming distance with two degrees to $(41n+1)$ for the PFSJS distance (18) with three degrees. For the vector-based similarity measures and the correlation measure, the number of operations ranges from $(16n+1)$ for the Dice similarity measure (22) with two degrees to $(24n)$ for Garg’s correlation measure (24) with three degrees. Finally, the proposed DFM also has a linear time complexity $\mathcal{O}(n)$ with $(15n)$ operations, which is quite analogous to the extant information measures with no additional computational cost.

### An MCGDM algorithm based on the proposed differential measure

Based on the aforementioned DFM, an algorithm for MCGDM problems can be summarized as follows:

**Step 1**: Identify the alternatives $\left({A}_{1}, {A}_{2},\dots ,{A}_{n}\right)$, the evaluation criteria $\left({C}_{1}, {C}_{2},\dots ,{C}_{m}\right)$, and assign the experts $\left({E}_{1}, {E}_{2},\dots ,{E}_{k}\right)$.

**Step 2**: (i) Form the decision matrices and determine the weights of the criteria

$$ \begin{aligned} & {\widetilde{\mathbf{D}}}_{\mathrm{p}}=\begin{array}{c}\\ \left[\begin{array}{ccc}\begin{array}{cc}{\widetilde{A}}_{11}^{p}& {\widetilde{A}}_{12}^{p}\\ {\widetilde{A}}_{21}^{p}& {\widetilde{A}}_{22}^{p}\end{array}& \cdots & \begin{array}{c}{\widetilde{A}}_{1m}^{p}\\ {\widetilde{A}}_{2m}^{p}\end{array}\\ \vdots & \ddots & \vdots \\ \begin{array}{cc}{\widetilde{A}}_{n1}^{p}& {\widetilde{A}}_{n2}^{p}\end{array}& \cdots & {\widetilde{A}}_{nm}^{p}\end{array}\right]\end{array},\; \\\\ & \quad \mathrm{and}\; {\widetilde{\mathbf{W}}}_{p}=\left[\begin{array}{cccc}{\widetilde{\mathrm{w}}}_{1}^{p}& {\widetilde{\mathrm{w}}}_{2}^{p}& \dots & {\widetilde{\mathrm{w}}}_{m}^{p}\end{array}\right], \end{aligned}$$

where ${\widetilde{A}}_{ij}^{p}$ denotes the PF rating of the *i*th alternative for the *j*th criterion as evaluated by the *p*th expert, and ${\widetilde{\mathrm{w}}}_{j}^{p}$ is the PF weight assigned by the expert to the *j*th criteria.

(ii) Normalize the decision matrices by replacing the rating of the cost criteria with its conjugate (12).

**Step 3**: Compute the aggregated decision matrix using (8), and then find the ideal rating for each criterion

$\widetilde{\mathbf{D}}=\left[{\widetilde{\mathrm{a}}}_{ij}\right], {\widetilde{\mathrm{a}}}_{ij}=\left(\sum_{p=1}^{k}{\omega }_{p}{\mu }_{{\widetilde{A}}_{ij}^{p}},\sum_{p=1}^{k}{\omega }_{p}{\upsilon }_{{\widetilde{A}}_{ij}^{p}}\right)$, ${\omega }_{p}$ is the weight of the *p*th expert,

25 $${\widetilde{IR}}_{j}=\left({\mu }_{j}^{+},{\upsilon }_{j}^{+}\right)=\left(\underset{i}{\mathrm{max}}{\mu }_{ij},\underset{i}{\mathrm{min}}{\upsilon }_{ij}\right).$$

**Step 4**: Find the DFM between the ideal rating and the rating of each alternative for a criterion, hence forming the differential matrix

26 $$\widetilde{\mathbb{D}}=\left[{\widetilde{\mathbbm{d}}}_{ij}\right],{\widetilde{\mathbbm{d}}}_{ij}= Diff\left({\widetilde{IR}}_{j},{\widetilde{a}}_{ij}\right).$$

**Step 5:** Compute the collective differential measure (CDFM) for each alternative

27 $$\widetilde{\mathbb{S}}=\left[{\widetilde{\mathbbm{s}}}_{i}\right],{\widetilde{\mathbbm{s}}}_{i}= \left(\sum_{j=1}^{m}{w}_{j}{\mu }_{{\widetilde{\mathbbm{d}}}_{ij}},\sum_{j=1}^{m}{w}_{j}{\upsilon }_{{\widetilde{\mathbbm{d}}}_{ij}} \right).$$

**Step 6:** Compute the score function of the CDFM and rank.

Using the score function (10), the crisp value of the CDFM of each alternative is calculated

$${{\mathbbm{s}}_{i}=Sc}_{ZX}\left({\widetilde{\mathbbm{s}}}_{i}\right).$$

The CDFM represents the total degree of superiority of the ideal ratings to the ratings of an alternative for the evaluation criteria. The smaller the degree of superiority of the ideal rating, the better the alternative is. Hence, the alternatives are arranged in ascending order and the alternative with the smallest value is the best.

## Determining the experts’ weights

Moving from a single expert to multiple experts became essential with the increasing complexity of the socio-economic environment as it is hard for a single expert to handle all the relevant aspects of a complex problem [83]. In group decision-making, it is almost impossible to have a homogeneous group of experts with similar attitudes, knowledge, and experience. The credibility of experts’ opinions and the effects of their opinion on the final decision should be considered. Ignoring the relative weights of the experts can lead to unreliable results. While studies on determining the weights of the criteria are numerous, studies on determining the weights of the experts are limited [84].

The experts’ weights are ordinarily determined subjectively. They are set by a supervisor or mutual evaluations of each other. This way of determining the experts’ weights has low credibility. Hence, objective methods that utilize quantitative techniques are employed to get the experts’ weight from a more objective perspective [85].

Zhang [58] proposed a method to determine the unknown weights of the experts based on the similarity of the individual expert’s opinion and the group’s opinion. The more the opinion of an expert is similar to the opinion of the group, the more important the expert is, and this expert should have a larger weight.

This study proposes a simple method to determine the unknown experts’ weights based on their consensus on the ranking of the alternatives. The ranking of each expert is obtained using the weighted sum method (WSM). Then, the correlation between the rankings of each two experts is calculated using Spearman’s correlation coefficient. The total correlation of an expert is calculated by adding his/her correlation with other experts. Finally, the total correlation of each expert is divided by the sum of the experts’ total correlation.

First, the judgments of each expert for an alternative for the evaluation criteria are aggregated using the Pythagorean fuzzy weighted averaging operator (8).

**Step 1**: Form the decision vector of each expert.

The ratings of the *p*th expert for the *i*th alternative are aggregated using (8)

28 $${\widetilde{\mathbf{V}}}_{\mathrm{p}}=\begin{array}{c}\boldsymbol{ }\boldsymbol{ }\boldsymbol{ }\boldsymbol{ }\boldsymbol{ }\boldsymbol{ }\\ \left[{\widetilde{\mathbf{v}}}_{i}^{p}\right]=\left[\begin{array}{c}\begin{array}{c}\sum_{j=1}^{m}{\widetilde{\mathrm{w}}}_{j}^{p}{\widetilde{A}}_{1j}^{p}\\ \sum_{j=1}^{m}{\widetilde{\mathrm{w}}}_{j}^{p}{\widetilde{A}}_{2j}^{p}\end{array}\\ \vdots \\ \sum_{j=1}^{m}{\widetilde{\mathrm{w}}}_{j}^{p}{\widetilde{A}}_{nj}^{p}\end{array}\right]\end{array}.$$

**Step 2:** Compute the score of the ratings for each alternative and rank

29 $${\mathbf{V}}_{\mathrm{p}}=\begin{array}{c}\boldsymbol{ }\boldsymbol{ }\boldsymbol{ }\boldsymbol{ }\boldsymbol{ }\boldsymbol{ }\\ \left[{\mathbf{v}}_{i}^{p}\right]=\left[\begin{array}{c}\begin{array}{c}{Sc}_{ZX}\left({\widetilde{\mathbf{v}}}_{1}^{p}\right)\\ {Sc}_{ZX}\left({\widetilde{\mathbf{v}}}_{2}^{p}\right)\end{array}\\ \vdots \\ {Sc}_{ZX}\left({\widetilde{\mathbf{v}}}_{n}^{p}\right)\end{array}\right]\end{array}.$$

The alternatives are ranked in descending order; the alternative with the largest score is the best option. Then, the rank of the *p*th expert is $\mathrm{R}\left({\mathrm{E}}_{p}\right)={A}_{p}^{1},{A}_{p}^{2},\dots ,{A}_{p}^{n}.$

**Step 3:** Compute the correlation between the rankings of each two experts using Spearman’s correlation coefficient.

The correlation between the ranking of the *p*th expert and each of the other experts is calculated using Spearman’s correlation coefficient

30 $$C\left({\mathrm{E}}_{p},{\mathrm{E}}_{h}\right)=1-\frac{6\sum {\Delta }_{ph}^{2}}{n\left({n}^{2}-1\right)},\mathrm{for} 1\le p,h\le k,p\ne h,$$

where $C\left({\mathrm{E}}_{p},{\mathrm{E}}_{h}\right)$ is the correlation between the ranking of the *p*th expert and the *h*th expert, and ${\Delta }_{ph}$ is the difference in the rank of the *i*th alternative as ordered by the *p*th expert and the *h*th expert.

**Step 4:** Calculate the total correlation of each expert.

The total correlation of the *p*th expert is the sum of his/her correlation with other experts

31 $$C\left({\mathrm{E}}_{p}\right)=\sum_{\begin{array}{c}h=1\\ h\ne p\end{array}}^{k}C\left({\mathrm{E}}_{p},{\mathrm{E}}_{h}\right).$$

**Step 5:** Determine the experts’ weights.

The total correlation of each expert is divided by the sum of the experts’ total correlation to obtain his/her weight

32 $$\omega \left({\mathrm{E}}_{p}\right)=\frac{C\left({\mathrm{E}}_{p}\right)}{\sum_{p=1}^{k}C\left({\mathrm{E}}_{p}\right)}.$$

**Fig. 1:**
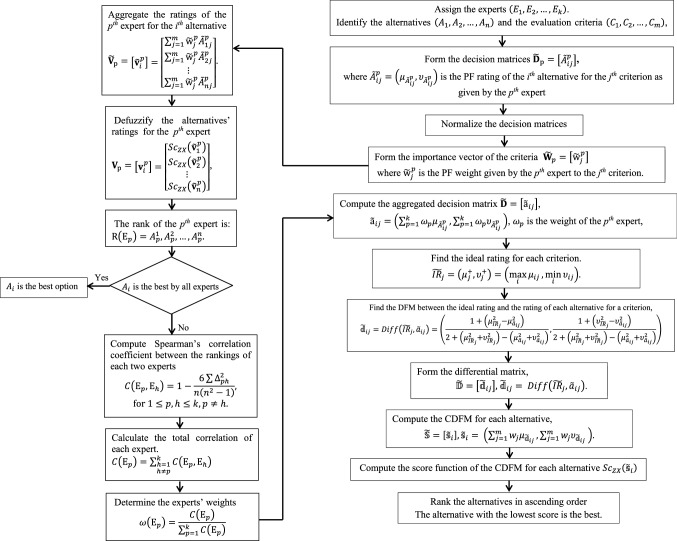
The proposed framework for MCGDM

The proposed MCGDM framework is shown in Fig. 1.

## Practical examples

In decision-making, one of the effective approaches to choosing an eligible alternative from several alternatives is its degree of similarity to the ideal rating. The highest similarity indicates the best alternative. Conversely, when using the differential measure, the lowest differential measure is an indication of the best alternative. In this section, the proposed MCGDM based on the differential measure is employed to handle two practical applications using Pythagorean fuzzy information. The first application is adapted from Huang et al. [50], the evaluation of solid-state drives (SSDs). The second application is adapted from Zhang [58], the selection of the best photovoltaic cell.

### The evaluation of SSDs

Nowadays, flash memories are the main storage technology for computers and mobile devices. Solid-state drives (SSDs) are widely used as secondary memory in modern computing systems instead of magnetic hard disk drives (HDDs). The performance of HDDs became stagnant due to the limitations in the seek time of actuator arms and the rotational speed of magnetic platters. On the other hand, SSDs do not have complex mechanical parts. This lowers both latency and failure rates than HDDs. Moreover, SSDs offer superior bandwidth, lower power consumption, higher random I/O performance, higher shock resistance, and improved system reliability compared to HDDs [86].

An enterprise wants to choose a type from five available types of solid-state drives (SSDs) denoted by $\left\{{A}_{1},{A}_{2},{A}_{3},{A}_{4},{A}_{5}\right\}$. The assessment is based on the following criteria: performance $\left({C}_{1}\right)$, reliability $\left({C}_{2}\right)$, capacity $\left({C}_{3}\right)$, form factor and connectors $\left({C}_{4}\right)$, battery life $\left({C}_{5}\right)$, speed $\left({C}_{6}\right)$, durability $\left({C}_{7}\right)$, and price $\left({C}_{8}\right)$. All the criteria are benefit ones except for the eighth criterion, the price is a cost criterion. The weights of the criteria are $w=\left(\mathrm{0.19,0.09,0.11,0.12,0.12,0.13,0.07,0.17}\right)$. An expert from the enterprise evaluates the SSDs for the given criteria using PFSs. The Pythagorean fuzzy decision matrix is given in Table 2 with the ideal rating.

**Table 2:** The decision matrix for the evaluation of SSDs

	${C}_{1}$	${C}_{2}$	${C}_{3}$	${C}_{4}$	${C}_{5}$	${C}_{6}$	${C}_{7}$	${C}_{8}$
${A}_{1}$	$\left(\mathrm{0.7,0.1}\right)$	$\left(\mathrm{0.5,0.2}\right)$	$\left(\mathrm{0.6,0.1}\right)$	$\left(\mathrm{0.6,0.1}\right)$	$\left(\mathrm{0.6,0.1}\right)$	$\left(\mathrm{0.7,0.1}\right)$	$\left(\mathrm{0.6,0.4}\right)$	$\left(\mathrm{0.6,0.2}\right)$
${A}_{2}$	$\left(\mathrm{0.6,0.1}\right)$	$\left(\mathrm{0.6,0.2}\right)$	$\left(\mathrm{0.6,0.3}\right)$	$\left(\mathrm{0.5,0.1}\right)$	$\left(\mathrm{0.5,0.3}\right)$	$\left(\mathrm{0.6,0.1}\right)$	$\left(\mathrm{0.6,0.4}\right)$	$\left(\mathrm{0.5,0.3}\right)$
${A}_{3}$	$\left(\mathrm{0.8,0.1}\right)$	$\left(\mathrm{0.6,0.1}\right)$	$\left(\mathrm{0.7,0.1}\right)$	$\left(\mathrm{0.6,0.2}\right)$	$\left(\mathrm{0.6,0.3}\right)$	$\left(\mathrm{0.7,0.1}\right)$	$\left(\mathrm{0.7,0.3}\right)$	$\left(\mathrm{0.5,0.2}\right)$
${A}_{4}$	$\left(\mathrm{0.3,0.6}\right)$	$\left(\mathrm{0.3,0.1}\right)$	$\left(\mathrm{0.5,0.6}\right)$	$\left(\mathrm{0.5,0.1}\right)$	$\left(\mathrm{0.4,0.2}\right)$	$\left(\mathrm{0.6,0.4}\right)$	$\left(\mathrm{0.2,0.5}\right)$	$\left(\mathrm{0.7,0.1}\right)$
${A}_{5}$	$\left(\mathrm{0.4,0.5}\right)$	$\left(\mathrm{0.3,0.2}\right)$	$\left(\mathrm{0.5,0.3}\right)$	$\left(\mathrm{0.4,0.2}\right)$	$\left(\mathrm{0.3,0.3}\right)$	$\left(\mathrm{0.4,0.5}\right)$	$\left(\mathrm{0.3,0.4}\right)$	$\left(\mathrm{0.6,0.1}\right)$
$IR$	$\left(\mathrm{0.8,0.1}\right)$	$\left(\mathrm{0.6,0.1}\right)$	$\left(\mathrm{0.7,0.1}\right)$	$\left(\mathrm{0.6,0.1}\right)$	$\left(\mathrm{0.6,0.1}\right)$	$\left(\mathrm{0.7,0.1}\right)$	$\left(\mathrm{0.7,0.3}\right)$	$\left(\mathrm{0.7,0.1}\right)$

Using the decision matrix, the differential measure between the ideal ratings and the ratings of each alternative for the criteria is calculated using (15). Then, the differential matrix is formed as given in Table 3.

**Table 3:** The differential matrix for the evaluation of the SSDs

	${C}_{1}$	${C}_{2}$	${C}_{3}$	${C}_{4}$	${C}_{5}$	${C}_{6}$	${C}_{7}$	${C}_{8}$
${A}_{1}$	$\left(\mathrm{0.53,0.47}\right)$	$\left(\mathrm{0.53,0.47}\right)$	$\left(\mathrm{0.53,0.47}\right)$	$\left(\mathrm{0.50,0.50}\right)$	$\left(\mathrm{0.50,0.50}\right)$	$\left(\mathrm{0.50,0.50}\right)$	$\left(\mathrm{0.55,0.45}\right)$	$\left(\mathrm{0.54,0.46}\right)$
${A}_{2}$	$\left(\mathrm{0.56,0.44}\right)$	$\left(\mathrm{0.51,0.49}\right)$	$\left(\mathrm{0.55,0.45}\right)$	$\left(\mathrm{0.53,0.47}\right)$	$\left(\mathrm{0.53,0.47}\right)$	$\left(\mathrm{0.55,0.45}\right)$	$\left(\mathrm{0.55,0.45}\right)$	$\left(\mathrm{0.57,0.43}\right)$
${A}_{3}$	$\left(\mathrm{0.50,0.50}\right)$	$\left(\mathrm{0.50,0.50}\right)$	$\left(\mathrm{0.50,0.50}\right)$	$\left(\mathrm{0.51,0.49}\right)$	$\left(\mathrm{0.51,0.49}\right)$	$\left(\mathrm{0.52,0.48}\right)$	$\left(\mathrm{0.50,0.50}\right)$	$\left(\mathrm{0.56,0.44}\right)$
${A}_{4}$	$\left(\mathrm{0.70,0.30}\right)$	$\left(\mathrm{0.56,0.44}\right)$	$\left(\mathrm{0.66,0.34}\right)$	$\left(\mathrm{0.53,0.47}\right)$	$\left(\mathrm{0.53,0.47}\right)$	$\left(\mathrm{0.55,0.45}\right)$	$\left(\mathrm{0.63,0.37}\right)$	$\left(\mathrm{0.50,0.50}\right)$
${A}_{5}$	$\left(\mathrm{0.67,0.34}\right)$	$\left(\mathrm{0.57,0.43}\right)$	$\left(\mathrm{0.57,0.43}\right)$	$\left(\mathrm{0.57,0.43}\right)$	$\left(\mathrm{0.57,0.43}\right)$	$\left(\mathrm{0.58,0.42}\right)$	$\left(\mathrm{0.60,0.40}\right)$	$\left(\mathrm{0.53,0.47}\right)$

The differential measures of each alternative are aggregated to get its collective differential measure. Then, using the score function (10), the CDFMs are defuzzified to get the total degree of superiority of the ideal ratings to the ratings of the alternatives for the evaluation criteria. Then, the alternatives are ranked in ascending order as given in Table 4. From Table 4, the final rank is ${A}_{3}>{A}_{1}>{A}_{2}>{A}_{4}>{A}_{5}$.

**Table 4:** The CDFM and ranking for the evaluation of the SSD problem

	Collective differential measure	$\mathrm{DoS}\left(\widetilde{IR},{\widetilde{A}}_{i}\right)$	Rank
${A}_{1}$	$\left(\mathrm{0.5229,0.4771}\right)$	0.0458	2
${A}_{2}$	$\left(\mathrm{0.5467,0.4533}\right)$	0.0934	3
${A}_{3}$	$\left(\mathrm{0.5138,0.4862}\right)$	0.0276	1
${A}_{4}$	$\left(\mathrm{0.5894,0.4106}\right)$	0.1788	4
${A}_{5}$	$\left(\mathrm{0.5922,0.4078}\right)$	0.1844	5

The result of the proposed method is compared with the results of other decision- making methods namely, the TOPSIS method proposed by Zhang and Xu [42], the TODIM approach developed by Ren et al. [44], the distance and similarity measure introduced by Zeng et al. [55], and the fuzzy weighted and ordered weighted aggregation operators introduced by Garg [87]. The rankings obtained by these methods [50] are given in Table 5.

**Table 5:** Ranking of SSDs using different methods

Method	Ranking results
Zhang and Xu’s method [42]	${A}_{3}{>A}_{1}>{A}_{2}{>A}_{5}{>A}_{4}$
Ren et al.’s method [44]	${A}_{3}{>A}_{1}>{A}_{2}>{A}_{5}{>A}_{4}$
Zeng et al.’s method [55]	${A}_{3}{>A}_{1}>{A}_{2}{>A}_{4}{>A}_{5}$
Garg’s method [87]	${A}_{3}{>A}_{2}>{A}_{1}{>A}_{5}{>A}_{4}$
Huang et al.’s method [50]	${A}_{3}{>A}_{2}>{A}_{1}{>A}_{5}{>A}_{4}$
Proposed method	${A}_{3}{>A}_{1}>{A}_{2}{>A}_{4}{>A}_{5}$

It can be seen from Table 5 that the proposed method derived the same optimal alternative as the previously used methods. The third alternative is the best option. The ranking list coincides with the results of Zeng et al.’s method. The first and second alternatives exchange places from one method to another, also the fourth and fifth alternatives.

### Photovoltaic cells

The current energy crisis in the world due to the scarcity of natural fuels in the earth’s crust has made great attention to the photovoltaic (PV) energy conversion technology [88]. Photovoltaic solar energy is the most promising alternative to conventional energy sources, providing a source of energy that is both sustainable and eco-friendly [89]. Photovoltaic systems generate electric energy directly through the solar light drive producing zero pollution [90]. They also have several advantages, e.g., noiseless operation, low maintenance, and high reliability [91]. In recent years, the development of emerging photovoltaic technologies has been subject to extensive research that focuses on increasing the efficiency and lifetime of these devices, combined with employing low-cost materials and processes [89]. These substantial efforts are coupled with remarkable efforts to find the best option and alternative depending on the required needs, utilizing multi-criteria decision methods, that have been kept side by side with this constant search [92].

A photovoltaic cell is a device that converts light energy into electricity by a process called the photovoltaic effect which is a known physical and chemical phenomenon. Several types of PV cells are categorized by generation. The first generation is the traditional type made of monocrystalline silicon or polycrystalline silicon and is most commonly used; they dominate the current solar energy market (over 90%). Attempts were made to develop new materials with low-cost fabrication technology due to the high-cost manufacturing of crystalline silicon. The second generation is the thin-film cells that were fabricated by cheaper technologies. The most commonly used materials are amorphous silicon (a-Si), nanocrystalline silicon (nc-Si), cadmium telluride (CdTe), and copper indium gallium selenide (CIGS). Yet, their performance is not higher than the first-generation cells. The third generation was designed to achieve both high efficiency and low cost. They include a variety of thin-film technologies, some of them generate electricity using organic materials, and others use inorganic substances, e.g., dye-sensitized cells (DSSCs), quantum dot-sensitized cells (QDSSCs), organic solar cells, and hybrid perovskite cells [88].

After the selection of the correct location to install a photovoltaic solar plant, it is required to select the kind of cell, from the various available PV cells, that optimizes the installation, e.g., increases production or efficiency, minimizes costs, and grants high maturity and reliability [92].

#### The selection of photovoltaic cells

It is required to select the best photovoltaic cell from the following alternatives: crystalline silicon $\left({A}_{1}\right)$, amorphous silicon $\left({A}_{2}\right)$, CdTe and CIGS $\left({A}_{3}\right)$, thin-film III–V with tracking systems $\left({A}_{4}\right)$, and organic and hybrid cells $\left({A}_{5}\right)$. These cells are assessed according to the following criteria: manufacturing cost $\left({C}_{1}\right)$, energy conversion efficiency $\left({C}_{2}\right)$, market share $\left({C}_{3}\right)$, emissions of greenhouse gases generated during the manufacturing process $\left({C}_{4}\right)$, and energy pay-back time $\left({C}_{5}\right)$. The second and third criteria are benefit criteria, and the rest are cost criteria. Three experts are involved in the evaluation process with weights $\omega =\left(0.3191, 0.3533, 0.3276\right)$. The weighting vector of the criteria is $w=\left(\mathrm{0.2,0.4,0.1,0.1,0.2}\right)$. The experts’ ratings of the alternatives for the assessment criteria are represented by PFSs. The decision matrices are formed and normalized to account for the cost criteria. The aggregated decision matrix is given directly in Table 6 using the ${\mathrm{PFWA}}_{Y}$ (8) together with the ideal rating $\left(\mathrm{IR}\right)$ using (25). The Pythagorean fuzzy decision matrices can be found in detail in Zhang [58].

**Table 6:** The aggregated decision matrix for the PV cells’ problem

	${C}_{1}$	${C}_{2}$	${C}_{3}$	${C}_{4}$	${C}_{5}$
${A}_{1}$	$\left(\mathrm{0.4259,0.8190}\right)$	$\left(\mathrm{0.7207,0.5897}\right)$	$\left(\mathrm{0.5242,0.7535}\right)$	$\left(\mathrm{0.3604,0.6224}\right)$	$\left(\mathrm{0.2948,0.5897}\right)$
${A}_{2}$	$\left(\mathrm{0.6552,0.4586}\right)$	$\left(\mathrm{0.8845,0.1966}\right)$	$\left(\mathrm{0.7862,0.2293}\right)$	$\left(\mathrm{0.2948,0.5242}\right)$	$\left(\mathrm{0.3931,0.4586}\right)$
${A}_{3}$	$\left(\mathrm{0.3276,0.5569}\right)$	$\left(\mathrm{0.5569,0.6224}\right)$	$\left(\mathrm{0.6552,0.3604}\right)$	$\left(\mathrm{0.3931,0.5569}\right)$	$\left(\mathrm{0.4586,0.4259}\right)$
${A}_{4}$	$\left(\mathrm{0.3931,0.7535}\right)$	$\left(\mathrm{0.6224,0.5242}\right)$	$\left(\mathrm{0.6224,0.1966}\right)$	$\left(\mathrm{0.2948,0.6224}\right)$	$\left(\mathrm{0.3604,0.6880}\right)$
${A}_{5}$	$\left(\mathrm{0.2621,0.6552}\right)$	$\left(\mathrm{0.7207,0.2621}\right)$	$\left(\mathrm{0.8518,0.2948}\right)$	$\left(\mathrm{0.5897,0.5897}\right)$	$\left(\mathrm{0.3604,0.6224}\right)$
$IR$	$\left(\mathrm{0.6552,0.4586}\right)$	$\left(\mathrm{0.8845,0.1966}\right)$	$\left(\mathrm{0.8518,0.1996}\right)$	$\left(\mathrm{0.5897,0.5242}\right)$	$\left(\mathrm{0.4586,0.4259}\right)$

Then, the differential measure between the ideal rating and the rating of an alternative for the evaluation criteria is calculated using (15), and the differential matrix is formed, as shown in Table 7.

**Table 7:** The differential measure matrix for the PV cells’ problem

	${C}_{1}$	${C}_{2}$	${C}_{3}$	${C}_{4}$	${C}_{5}$
${A}_{1}$	$\left(\mathrm{0.6981,0.3019}\right)$	$\left(\mathrm{0.6464,0.3536}\right)$	$\left(\mathrm{0.7549,0.2451}\right)$	$\left(\mathrm{0.5785,0.4215}\right)$	$\left(\mathrm{0.5740,0.4260}\right)$
${A}_{2}$	$\left(\mathrm{0.5000,0.5000}\right)$	$\left(\mathrm{0.5000,0.5000}\right)$	$\left(\mathrm{0.5290,0.4710}\right)$	$\left(\mathrm{0.5577,0.4423}\right)$	$\left(\mathrm{0.5209,0.4791}\right)$
${A}_{3}$	$\left(\mathrm{0.5949,0.4051}\right)$	$\left(\mathrm{0.6933,0.3067}\right)$	$\left(\mathrm{0.5879,0.4121}\right)$	$\left(\mathrm{0.5530,0.4470}\right)$	$\left(\mathrm{0.5000,0.5000}\right)$
${A}_{4}$	$\left(\mathrm{0.6648,0.3352}\right)$	$\left(\mathrm{0.6462,0.3538}\right)$	$\left(\mathrm{0.5723,0.4277}\right)$	$\left(\mathrm{0.5869,0.4131}\right)$	$\left(\mathrm{0.6041,0.3959}\right)$
${A}_{5}$	$\left(\mathrm{0.6353,0.3647}\right)$	$\left(\mathrm{0.5656,0.4344}\right)$	$\left(\mathrm{0.5124,0.4876}\right)$	$\left(\mathrm{0.5189,0.4811}\right)$	$\left(\mathrm{0.5764,0.4236}\right)$

The differential measures of each alternative are aggregated to get its collective differential measure. Then, the score function (10) is computed and the alternatives are ranked as given in Table 8.

**Table 8:** The collective differential measure and ranking for PV cells’ problem

	Collective differential measure	$\mathrm{DoS}\left(\widetilde{\mathrm{IR}},{\widetilde{A}}_{i}\right)$	Rank
${A}_{1}$	$\left(\mathrm{0.6463,0.3537}\right)$	0.2927	5
${A}_{2}$	$\left(\mathrm{0.5128,0.4872}\right)$	0.0257	1
${A}_{3}$	$\left(\mathrm{0.6104,0.3896}\right)$	0.2208	3
${A}_{4}$	$\left(\mathrm{0.6282,0.3718}\right)$	0.2563	4
${A}_{5}$	$\left(\mathrm{0.5717,0.4283}\right)$	0.1434	2

It is clear from Table 8 that the final rank is ${A}_{2}>{A}_{5}>{A}_{3}>{A}_{4}>{A}_{1}$. Hence, the best photovoltaic cell is ${A}_{2}$ (amorphous silicon).

Zhang [58] introduced an approach for multi-criteria group decision-making (MCGDM) based on similarity measures and newly developed aggregation operators. Biswas and Sarkar [60] also proposed a method for MCGDM through similarity measures based on point operators. They utilized these similarity measures to develop two new aggregation operators, namely, the Pythagorean fuzzy-dependent averaging operator and the Pythagorean fuzzy-dependent geometric operator. These operators are employed to aggregate the experts’ evaluations. The results of the proposed method are compared with the ranking obtained using the method of Zhang [58] and the method of Biswas and Sarkar [60]. The comparison is given in Table 9.

**Table 9:** Ranking the PV cells using different methods

Method	Ranking results
Zhang’s method [58]	${A}_{2}{>A}_{5}>{A}_{4}{>A}_{3}{>A}_{1}$
Biswas and Sarkar’s method [60]	${A}_{2}{>A}_{5}>{A}_{3}{>A}_{4}{>A}_{1}$
Proposed method	${A}_{2}{>A}_{5}>{A}_{3}{>A}_{4}{>A}_{1}$

From Table 9, the ranking obtained by the proposed method coincides with the ranking of Biswas and Sarkar [60]. It is also evident that the ranking of the proposed method is almost similar to the results of Zhang [58]. There is a slight difference in the moderately performing technologies, while the two top technologies and worst technology remain unchanged.

#### Applying the proposed weighting technique

In the previous PV cell selection problem, the experts’ weights can be determined using the proposed technique. The steps are illustrated as follows.

**Step 1**: Form the decision vector of each expert (28) using (8)

$${\widetilde{\mathbf{V}}}_{1}=\left[\begin{array}{c}\left(\mathrm{0.59,0.67}\right)\\ \left(\mathrm{0.73,0.39}\right)\\ \left(\mathrm{0.39,0.54}\right)\\ \left(\mathrm{0.57,0.54}\right)\\ \left(\mathrm{0.51,0.54}\right)\end{array}\right],\boldsymbol{ }{\widetilde{\mathbf{V}}}_{2}=\left[\begin{array}{c}\left(\mathrm{0.48,0.68}\right)\\ \left(\mathrm{0.67,0.32}\right)\\ \left(\mathrm{0.53,0.56}\right)\\ \left(\mathrm{0.54,0.59}\right)\\ \left(\mathrm{0.62,0.44}\right)\end{array}\right],\; \mathrm{and}\, {\widetilde{\mathbf{V}}}_{3}=\left[\begin{array}{c}\left(\mathrm{0.52,0.65}\right)\\ \left(\mathrm{0.65,0.33}\right)\\ \left(\mathrm{0.56,0.54}\right)\\ \left(\mathrm{0.39,0.64}\right)\\ \left(\mathrm{0.517,0.39}\right)\end{array}\right].$$

**Step 2:** Compute the score of each alternative (29) using (10) and rank the alternatives

$${\mathbf{V}}_{1}=\begin{array}{c}\boldsymbol{ }\boldsymbol{ }\boldsymbol{ }\boldsymbol{ }\boldsymbol{ }\boldsymbol{ }\\ \left[\begin{array}{c}-0.1008\\ 0.3808\\ -0.1395\\ 0.1649\\ -0.0315\end{array}\right]\end{array},{\mathbf{V}}_{2}=\begin{array}{c}\boldsymbol{ }\boldsymbol{ }\boldsymbol{ }\boldsymbol{ }\boldsymbol{ }\boldsymbol{ }\\ \left[\begin{array}{c}-0.2320\\ 0.3465\\ -0.0327\\ -0.0565\\ 0.1908\end{array}\right]\end{array},\mathrm{and }\, {\mathbf{V}}_{3}=\begin{array}{c}\boldsymbol{ }\boldsymbol{ }\boldsymbol{ }\boldsymbol{ }\boldsymbol{ }\boldsymbol{ }\\ \left[\begin{array}{c}-0.1521\\ 0.3201\\ 0.0220\\ -0.2575\\ 0.1728\end{array}\right]\end{array}.$$

From the scores, the ranking of each expert is

$$\mathrm{R}\left({\mathrm{E}}_{1}\right)=\begin{array}{c}\boldsymbol{ }\boldsymbol{ }\boldsymbol{ }\boldsymbol{ }\boldsymbol{ }\boldsymbol{ }\\ \left[\begin{array}{c}{A}_{2}\\ {A}_{4}\\ {A}_{5}\\ {A}_{1}\\ {A}_{3}\end{array}\right]\end{array},\mathrm{R}\left({\mathrm{E}}_{2}\right)=\begin{array}{c}\boldsymbol{ }\boldsymbol{ }\boldsymbol{ }\boldsymbol{ }\boldsymbol{ }\boldsymbol{ }\\ \left[\begin{array}{c}{A}_{2}\\ {A}_{5}\\ {A}_{3}\\ {A}_{4}\\ {A}_{1}\end{array}\right]\end{array},\; \mathrm{and}\mathrm{R}\left({\mathrm{E}}_{3}\right)=\begin{array}{c}\boldsymbol{ }\boldsymbol{ }\boldsymbol{ }\boldsymbol{ }\boldsymbol{ }\boldsymbol{ }\\ \left[\begin{array}{c}{A}_{2}\\ {A}_{5}\\ {A}_{3}\\ {A}_{1}\\ {A}_{4}\end{array}\right]\end{array}.$$

**Step 3:** Compute the correlation between the experts’ rankings using Spearman’s correlation coefficient (30). The results are given in Table 10.

**Table 10:** Correlation between the ranking of the experts

Alternatives	$\mathrm{R}({\mathrm{E}}_{1})$	$\mathrm{R}({\mathrm{E}}_{2})$	${\Delta }_{12}^{2}$	$\mathrm{R}({\mathrm{E}}_{1})$	$\mathrm{R}({\mathrm{E}}_{3})$	${\Delta }_{13}^{2}$	$\mathrm{R}({\mathrm{E}}_{2})$	$\mathrm{R}({\mathrm{E}}_{3})$	${\Delta }_{23}^{2}$
${A}_{1}$	4	5	1	4	4	0	5	4	1
${A}_{2}$	1	1	0	1	1	0	1	1	0
${A}_{3}$	5	3	4	5	3	4	3	3	0
${A}_{4}$	2	4	4	2	5	9	4	5	1
${A}_{5}$	3	2	1	3	2	1	2	2	0
	$C\left({\mathrm{E}}_{1},{\mathrm{E}}_{2}\right)=0.5$			$C\left({\mathrm{E}}_{1},{\mathrm{E}}_{3}\right)=0.3$			$C\left({\mathrm{E}}_{2},{\mathrm{E}}_{3}\right)=0.9$		

**Step 4:** Calculate the total correlation of each expert (25)

$$C\left({\mathrm{E}}_{1}\right)=C\left({\mathrm{E}}_{1},{\mathrm{E}}_{2}\right)+C\left({\mathrm{E}}_{1},{\mathrm{E}}_{3}\right)=0.8,$$

$$C\left({\mathrm{E}}_{2}\right)=C\left({\mathrm{E}}_{1},{\mathrm{E}}_{2}\right)+C\left({\mathrm{E}}_{2},{\mathrm{E}}_{3}\right)=1.4,$$

$$C\left({\mathrm{E}}_{3}\right)=C\left({\mathrm{E}}_{1},{\mathrm{E}}_{3}\right)+C\left({\mathrm{E}}_{2},{\mathrm{E}}_{3}\right)=1.2.$$

**Step 5:** Determine the experts’ weights (26)

$$\omega \left({\mathrm{E}}_{1}\right)=\frac{0.8}{3.4}=0.24, \omega \left({\mathrm{E}}_{2}\right)=\frac{1.4}{3.4}=0.41,\omega \left({\mathrm{E}}_{3}\right)=\frac{1.2}{3.4}=0.35.$$

When the PV cell selection problem is resolved using the weights obtained by the proposed technique, the results obtained are given in Table 11. The ranking obtained is consistent with the previously obtained results.

**Table 11:** The collective differential measure and ranking for PV cells using the proposed weighting technique

	CDFM	$\mathrm{DoS}\left(\widetilde{\mathrm{IR}},{\widetilde{A}}_{i}\right)$	Rank
${A}_{1}$	$\left(\mathrm{0.6682,0.3318}\right)$	0.3364	5
${A}_{2}$	$\left(\mathrm{0.5145,0.4855}\right)$	0.0291	1
${A}_{3}$	$\left(\mathrm{0.6248,0.3752}\right)$	0.2496	3
${A}_{4}$	$\left(\mathrm{0.6460,0.3540}\right)$	0.2920	4
${A}_{5}$	$\left(\mathrm{0.5813,0.4187}\right)$	0.1627	2

It is worth noting that this technique can yield the best alternative while determining the experts’ weights. Having obtained the ranking of the alternatives for each expert, if an alternative is in the first position by all experts, then it is the best choice, and no need to proceed unless a complete ranking list is required. In the previous example, the alternative ${A}_{2}$ is ranked first by the three experts. Then, it is the optimal alternative.

## The sensitivity analysis

This section aims to explore the reliability and stability of the proposed MCGDM method. Wang and Triantaphyllou [93] came up with three procedures to test the performance of different multi-criteria decision-making methods by changing some of the problem’s data.

- *Procedure 1* The best alternative does not change when a non-optimal alternative is replaced by another worse alternative, keeping the relative importance of each decision criterion unchanged.
- *Procedure 2 *When the decision problem is decomposed into smaller subproblems, the rankings of alternatives should follow the transitivity property.
- *Procedure 3* When the rankings of these subproblems are combined, the new overall ranking of the alternatives should be identical to the original overall ranking of the undecomposed problem.

The previously two solved problems are examined using these procedures as follows.

In the SSD evaluation problem, to apply Procedure 1, one of the non-optimal alternatives is replaced by a worse alternative. Since $\left({A}_{2}\right)$ is a non-optimal alternative, then it is replaced by an alternative whose ratings are the conjugate of the ratings of ${A}_{2},$ whenever the support is greater than the opposition, to guarantee being worse. The ranking obtained is given in Table 12.

**Table 12:** The collective differential measure and ranking for the modified SSD problem

	Collective differential measure	$\mathrm{DoS}\left(\widetilde{\mathrm{IR}},{\widetilde{A}}_{i}\right)$	Rank
${A}_{1}$	$\left(\mathrm{0.5229,0.4771}\right)$	0.0458	2
${A}_{2}^{^{\prime}}$	$\left(\mathrm{0.6688,0.3312}\right)$	0.3376	5
${A}_{3}$	$\left(\mathrm{0.5138,0.4862}\right)$	0.0276	1
${A}_{4}$	$\left(\mathrm{0.5894,0.4106}\right)$	0.1788	3
${A}_{5}$	$\left(\mathrm{0.5922,0.4078}\right)$	0.1844	4

From Table 12, the ranking is ${A}_{3}{>A}_{1}>{A}_{4}{>A}_{5}>{A}_{2}^{^{\prime}}$. Hence, the best alternative ${A}_{3}$ remains unchanged.

To apply Procedure 2 and Procedure 3, three subproblems are solved. Each sub-problem consists of three alternatives, i.e., two alternatives are omitted each time from the original problem. The results obtained are given in Table 13.

**Table 13:** The ranking of subproblems of the SSD selection problem

	Subproblem 1		Subproblem 2		Subproblem 3	
	CDFM	$\mathrm{DoS}\left(\widetilde{\mathrm{IR}},{\widetilde{A}}_{i}\right)$	CDFM	$\mathrm{DoS}\left(\widetilde{\mathrm{IR}},{\widetilde{A}}_{i}\right)$	CDFM	$\mathrm{DoS}\left(\widetilde{\mathrm{IR}},{\widetilde{A}}_{i}\right)$
${A}_{1}$	$\left(\mathrm{0.5177,0.4823}\right)$	0.0354	–	–	$\left(\mathrm{0.5164,0.4836}\right)$	0.0328
${A}_{2}$	–	–	$\left(\mathrm{0.5148,0.4852}\right)$	0.0296	$\left(\mathrm{0.5407,0.4593}\right)$	0.0814
${A}_{3}$	$\left(\mathrm{0.5091,0.4909}\right)$	0.0183	–	–	$\left(\mathrm{0.5078,0.4922}\right)$	0.0157
${A}_{4}$	–	–	$\left(\mathrm{0.5617,0.4383}\right)$	0.1234	–	–
${A}_{5}$	$\left(\mathrm{0.5870,0.4130}\right)$	0.1740	$\left(\mathrm{0.5652,0.4348}\right)$	0.1304	–	–
	${A}_{3}>{A}_{1}>{A}_{5}$		${A}_{2}>{A}_{4}>{A}_{5}$		${A}_{3}>{A}_{1}>{A}_{2}$	

From Table 13, the rankings of the alternatives from the subproblems follow the transitivity property. From sub-problem 3, we have ${A}_{1}>{A}_{2}.$ From sub-problem 2, we have ${A}_{2}>{A}_{5}$. This indicates that ${A}_{1}>{A}_{5}$, which is ascertained by sub-problem 1. Combining the rankings of these subproblems, the new overall ranking is ${A}_{3}{>A}_{1}>{A}_{2}{>A}_{4}{>A}_{5}$ which is identical to the overall ranking of the original problem.

Regarding the PV cells’ problem, the alternative $\left({A}_{3}\right)$ is replaced by another alternative $\left({A}_{3}^{^{\prime}}\right)$ whose ratings are replaced with the conjugate of the ratings of $\left({A}_{3}\right)$ whenever the support is greater than the opposition. The results are given in Table 14.

**Table 14:** The collective differential measure and ranking for the modified PV cells’ problem

	Collective differential measure	$\mathrm{DoS}\left(\widetilde{\mathrm{IR}},{\widetilde{A}}_{i}\right)$	Rank
${A}_{1}$	$\left(\mathrm{0.6421,0.3579}\right)$	0.2843	4
${A}_{2}$	$\left(\mathrm{0.5087,0.4913}\right)$	0.0173	1
${A}_{3}^{^{\prime}}$	$\left(\mathrm{0.6439,0.3561}\right)$	0.2877	5
${A}_{4}$	$\left(\mathrm{0.6237,0.3763}\right)$	0.2474	3
${A}_{5}$	$\left(\mathrm{0.5673,0.4326}\right)$	0.1347	2

From Table 14, the ranking is ${A}_{2}{>A}_{5}>{A}_{4}{>A}_{1}>{A}_{3}^{^{\prime}}$, and the best alternative is unchanged.

To apply Procedure 2 and procedure 3, six subproblems are solved. First, three subproblems are solved each having two alternatives. Then, another three subproblems are solved each consisting of three alternatives. The results obtained are given in Table 15.

**Table 15:** The ranking of subproblems of the PV cells

	Subproblem 1		Subproblem 2		Subproblem 3	
	CDFM	$\mathrm{DoS}\left(\widetilde{\mathrm{IR}},{\widetilde{A}}_{i}\right)$	CDFM	$\mathrm{DoS}\left(\widetilde{\mathrm{IR}},{\widetilde{A}}_{i}\right)$	CDFM	$\mathrm{DoS}\left(\widetilde{\mathrm{IR}},{\widetilde{A}}_{i}\right)$
${A}_{3}$	$\left(\mathrm{0.5678,0.4322}\right)$	0.1356	$\left(\mathrm{0.5240,0.4760}\right)$	0.0481	–	–
${A}_{4}$	–	–	$\left(\mathrm{0.5360,0.4640}\right)$	0.0720	$\left(\mathrm{0.610,0.4390}\right)$	0.1649
${A}_{5}$	$\left(\mathrm{0.5245,0.4755}\right)$	0.0489	–	–	$\left(\mathrm{0.5054,0.4946}\right)$	0.0107
	${A}_{5}>{A}_{3}$		${A}_{3}>{A}_{4}$		${A}_{5}>{A}_{4}$	

	Subproblem 4		Subproblem 5		Subproblem 6	
	CDFM	$\mathrm{DoS}\left(\widetilde{\mathrm{IR}},{\widetilde{A}}_{i}\right)$	CDFM	$\mathrm{DoS}\left(\widetilde{\mathrm{IR}},{\widetilde{A}}_{i}\right)$	CDFM	$\mathrm{DoS}\left(\widetilde{\mathrm{IR}},{\widetilde{A}}_{i}\right)$
${A}_{1}$	–	–	$\left(\mathrm{0.5680,0.4320}\right)$	0.1360	–	–
${A}_{2}$	$\left(\mathrm{0.5125,0.4875}\right)$	0.0250	–	–	–	–
${A}_{3}$	$\left(\mathrm{0.6100,0.3900}\right)$	0.2200	$\left(\mathrm{0.5367,0.4633}\right)$	0.0733	$\left(\mathrm{0.5728,0.4272}\right)$	0.1456
${A}_{4}$	–	–	$\left(\mathrm{0.5539,0.4461}\right)$	0.1079	$\left(\mathrm{0.5824,0.4176}\right)$	0.1649
${A}_{5}$	$\left(\mathrm{0.5714,0.4286}\right)$	0.1427	–	–	$\left(\mathrm{0.5248,0.4752}\right)$	0.0496
	${A}_{2}>{A}_{5}>{A}_{3}$		${A}_{3}>{A}_{4}>{A}_{1}$		${A}_{5}>{A}_{3}>{A}_{4}$	

The transitivity property is demonstrated in Table 15. The overall ranking can be obtained as ${A}_{2}{>A}_{5}>{A}_{3}{>A}_{4}{>A}_{1}$ which is the same ranking as the undecomposed problem.

From the previous illustrations, the proposed method successfully fulfilled the evaluation procedures. Therefore, it can be concluded that it is consistent and stable.

## Conclusion and discussion

This article proposed the concept of the DFM for the discrimination of PFSs. It is a preference relation between two PFSs by virtue of position in the attribute space and according to the closeness of their membership and non-membership degrees. The main objective of the DFM is to overcome the deficiencies that might arise in the existing measures. The drawbacks in the extant distance measures and similarity measures result from handling the membership (support) and non-membership degree (opposition) equally, although each direction has a different implication. A DFM treats the changes in the parameters of a Pythagorean fuzzy assessment differently. An increment in the support direction is considered a positive step, while an increment in the opposition direction is considered a negative step. Two PFSs are classified as identical, equivalent, superior, or inferior to one another giving the degree of superiority or inferiority. An MCGDM method is proposed based on the introduced DFM and a new technique for computing the weights of the experts is developed.

Two practical problems were solved to illustrate the method. The results of the proposed method are compared with the results of some extant MCDM methods. The main aim of the comparison is to verify and demonstrate the applicability of the method. None of the MCDM methods can be considered better than the others; there is no worst or best technique. Different results can be obtained through distinct methods, even if the processed data and information are the same. We can find a method that is most relevant in a given particular situation [94]. The proposed method would be superior whenever the MCDM methods based on distance or similarity measures are affected by the defects previously mentioned leading to incorrect results.

The first example is the evaluation of solid-state drives. The result of the proposed method is compared with the results of other decision-making methods, namely, the TOPSIS method proposed by Zhang and Xu [42], the TODIM approach developed by Ren et al. [44], the distance and similarity measures introduced by Zeng et al. [55], the fuzzy weighted and ordered weighted aggregation operators introduced by Garg [87], and the PF-MULTIMOORA proposed by Huang et al. [50]. The best option obtained by the applied methods is the same. The ranking list obtained by the proposed method coincides with the results of Zeng et al.’s method, with slight changes from other ranking lists.

The second example is the selection of the best photovoltaic cell. The result of the proposed method is compared with the approach of Zhang [58] and the method of Biswas and Sarkar [60]. The three methods derived the same best alternative. The ranking list of the proposed method coincides with the ranking list of Biswas and Sarkar [60] and is almost similar to the ranking list of Zhang [58].

A sensitivity analysis was conducted to examine the performance of the proposed MCGDM method. Various MCDM methods have different stabilities. For example, the stability of the MULTIMOORA method is good; for the TOPSIS method, it is poor; for the VIKOR and the ELECTRE methods, it is medium [50]. The proposed method is examined using the test procedures of Wang and Triantaphyllou [93]. The method successfully passed the three test procedures indicating its high stability and practicality.

Finally, a new technique for determining the weights of the experts is developed based on Spearman’s correlation coefficient and applied to the photovoltaic cell problem. The obtained experts’ weights are slightly different from the weights obtained by Zhang [58]. Yet, the relative importance of the experts is the same, and when the problem is resolved using the proposed weights, the same solution is attained.

Practically, the proposed DFM can be applied in different applications, e.g., image processing, pattern recognition, machine learning, information retrieval, medical diagnosis, and decision-making. In the decision-making process, the proposed framework can eliminate the biases that may arise due to assigning subjective weights to the decision-makers that can lead to unreliable results in many cases [47]. The framework provides objective weights for the decision-makers to overcome the undesirable effect of subjective weights. Therefore, for managers, the proposed framework can lead to better decisions. It is expected that the developed method can be successfully applied in any GDM environment, such as industrial engineering, and business management.

The proposed framework has the following limitations:

- PFSs are designed to address only two components of human perception independently, namely, preference and non-preference represented by the membership and non-membership degrees. The third component of human perception, hesitation, is handled as a dependent component. Actually, hesitation can be handled as an independent component with the previous two degrees. This is not covered by the frame of PFSs.
- The proposed differential measure lacks symmetry, i.e., $\mathrm{Diff}\left(\widetilde{A},\widetilde{B}\right)\ne {\mathrm{Diff}}^{c}\left(\widetilde{B},\widetilde{A}\right)$, in general.

Hence, further investigations are required to propose a symmetric differential measure in future work. The concept will also be developed in the spherical fuzzy environment which encompasses three independent degrees, membership, non-membership, and hesitation.

## Data Availability

Not applicable.
